# The specificity of *Babesia*-tick vector interactions: recent advances and pitfalls in molecular and field studies

**DOI:** 10.1186/s13071-021-05019-3

**Published:** 2021-09-28

**Authors:** Anna Bajer, Dorota Dwużnik-Szarek

**Affiliations:** Department of Eco-Epidemiology of Parasitic Diseases, Institute of Developmental Biology and Biomedical Sciences, Faculty of Biology, University of Warsaw, Miecznikowa 1, 02-096 Warsaw, Poland

**Keywords:** Piroplasm, Polymerase chain reaction, Sequencing, Phylogenetic analysis, Ticks

## Abstract

**Background:**

*Babesia* spp. are protozoan parasites of great medical and veterinary importance, especially in the northern Hemisphere. Ticks are known vectors of *Babesia* spp., although some *Babesia-*tick interactions have not been fully elucidated.

**Methods:**

The present review was performed to investigate the specificity of *Babesia*-tick species interactions that have been identified using molecular techniques in studies conducted in the last 20 years under field conditions. We aimed to indicate the main vectors of important *Babesia* species based on published research papers (*n* = 129) and molecular data derived from the GenBank database.

**Results:**

Repeated observations of certain *Babesia* species in specific species and genera of ticks in numerous independent studies, carried out in different areas and years, have been considered epidemiological evidence of established *Babesia*-tick interactions. The best studied species of ticks are *Ixodes ricinus*, *Dermacentor reticulatus* and *Ixodes scapularis* (103 reports, i.e. 80% of total reports). Eco-epidemiological studies have confirmed a specific relationship between *Babesia microti* and *Ixodes ricinus*, *Ixodes persulcatus*, and *Ixodes scapularis* and also between *Babesia canis* and *D. reticulatus*. Additionally, four *Babesia* species (and one genotype), which have different deer species as reservoir hosts, displayed specificity to the *I. ricinus* complex. Eco-epidemiological studies do not support interactions between a high number of *Babesia* spp. and *I. ricinus* or *D. reticulatus*. Interestingly, pioneering studies on other species and genera of ticks have revealed the existence of likely new *Babesia* species, which need more scientific attention. Finally, we discuss the detection of *Babesia* spp. in feeding ticks and critically evaluate the data on the role of the latter as vectors.

**Conclusions:**

Epidemiological data have confirmed the specificity of certain *Babesia-*tick vector interactions. The massive amount of data that has been thus far collected for the most common tick species needs to be complemented by more intensive studies on *Babesia* infections in underrepresented tick species.

**Graphical abstract:**

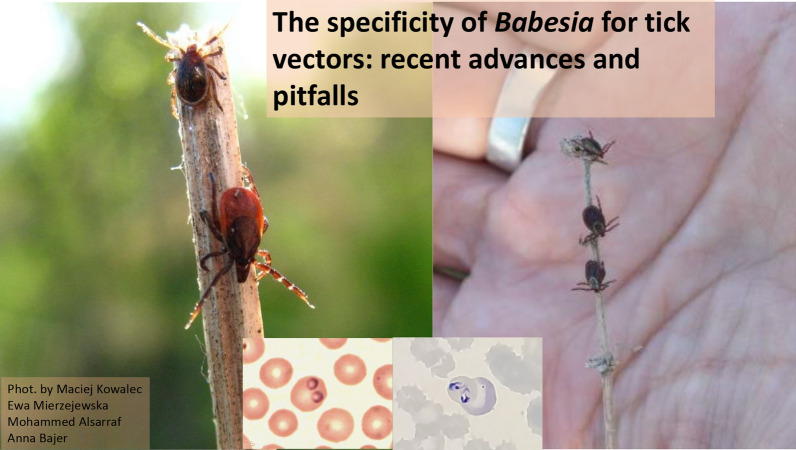

**Supplementary Information:**

The online version contains supplementary material available at 10.1186/s13071-021-05019-3.

## Background

*Babesia* spp. are protozoan parasites of great medical and veterinary importance, especially in the northern Hemisphere [1, 2]. Amongst the many *Babesia* species that infect animals, *Babesia bovis* and *Babesia bigemina* are notable for the significant economic losses they cause in the cattle industry worldwide [3], and several *Babesia* species (i.e. *Babesia canis*, *Babesia rossi*, *Babesia vogeli*, *Babesia gibsoni*, *Babesia conradae* and *Babesia vulpes*) may cause serious health problem in dogs [4–6]. There is increasing interest in babesiosis in humans due to the rising number of cases in the USA [2, 7], Canada [8] and China [7, 9]. In the USA alone, the cumulative number of cases of babesiosis in humans from 2006 to 2018 was estimated to be between 20,000 and 24,000 [7]. In Canada, over 1100 human cases, mostly due to *Babesia duncani*, have been recently reported [7, 8]. In China, over 125 cases have been reported, including 58 due to a *Babesia crassa*-like novel pathogen [7, 9–12].

Hard ticks are the vectors of *Babesia* parasites, which are emerging tick-borne pathogens [1, 13]. In a recent review/meta-analysis on *Babesia* spp. prevalence in questing ticks, the estimated global prevalence was 2.1% [14]. However, this prevalence was calculated jointly for 19 different *Babesia* species and 23 tick species.

In the life cycle of piroplasms, obligate intracellular parasites that belong to the phylum Apicomplexa [15, 16], ticks play a pivotal role as definitive hosts, in which sexual reproduction of the parasite (gametogony) occurs, followed by asexual amplification (sporogony), resulting in life stages invasive for vertebrate hosts (sporozoites). As highly specialized intracellular parasites, *Babesia* are believed to display a high specificity for both tick vectors and vertebrate hosts [1]. However, humans may be an example of broadened/disrupted host specificity for *Babesia*, as there is no human-specific *Babesia* species and babesiosis in humans is caused by several zoonotic species, including *Babesia microti*, *Babesia divergens*, *Babesia venatorum*, *Babesia duncani* and *Babesia crassa*-like [2, 7, 9].

Interestingly, a single tick species may act as a specific vector for several species of *Babesia*, e.g. *Ixodes ricinus* has already been indicated as a presumptive vector for at least nine species of *Babesia*, *Ixodes persulcatus* for five, and *Dermacentor reticulatus* for six [14]. However, this phenomenon is not contradictory to the specificity of certain *Babesia* sp.-tick vector interactions. In addition, the main vectors for many important *Babesia* species, including *B. conradae*, *B. duncani* and *B. crassa*-like, have yet to be identified.

This review was carried out to investigate the specificity of *Babesia*-tick interactions that have been identified using molecular techniques in studies performed over the past 20 years under field conditions. Based on published research papers and molecular data derived from the GenBank database (Additional file 1: Text S1), we indicate the main vectors for important *Babesia* species. Finally, we discuss the detection of *Babesia* spp. in feeding ticks and critically evaluate the data on the role of the latter as vectors.

## Proving the specificity of a *Babesia*-tick vector interaction

The first records of babesiosis in cattle (also termed Texas fever or redwater disease) and dogs (also termed malignant jaundice and bilious fever) are from the end of the nineteenth century (reviewed in [3, 4, 6]). At that time, a classical approach to identifying the etiological agent and vector of a disease was based on experimental infection under controlled conditions by injecting blood from an infected dog into a naïve one, or through the infestation of naïve animals with a suspected tick vector [6]. For canine babesiosis, early research carried out from the 1890s to the 1930s showed that there were three distinct vector-specific parasites in different regions of the world. Interestingly, this knowledge was overlooked for the next 50 years, and only at the end of twentieth century was the ‘*Babesia canis’* complex of species divided into three distinct vector-specific species: *Babesia canis*, with the ornate dog tick *D. reticulatus* as its vector; *Babesia rossi*, with *Haemaphysalis elliptica* as its vector; and *Babesia vogeli*, with the brown dog tick *Rhipicephalus sanguineus* sensu lato (s.l.) as its vector [6, 17, 18].

In recent years, the use of novel laboratory/molecular biology techniques allowing for the identification of genetic material of pathogens/endosymbionts in ticks collected from humans, domestic animals, wildlife, or the environment, has resulted in an enormous increase in new data on tick-microorganism interactions. This rapidly growing amount of new information for various tick-borne pathogens, including *Babesia*, presents challenges, including how the detection of the genetic material of pathogens in ticks should best be interpreted [19]. A review focused on the vector competence of hard ticks and *Borrelia burgdorferi* sensu lato spirochetes [20] underlined the pitfalls of concluding vector competence based only on the detection of pathogen DNA in ticks, i.e. without complementary experimental studies.

A well-established, experimental approach to conclusively prove vector competence should encompass three distinct processes: the acquisition of a piroplasm by uninfected ticks feeding on an infected experimental host (or on infected blood in in vitro experiments); the maintenance of the piroplasm through the moult to the next life stadium (transstadial transmission); and, finally, transmission of the piroplasm to naïve hosts during a subsequent blood meal (based on [20]). A tick species should not be considered a competent vector of *Babesia* spp. unless all three of these processes have been experimentally demonstrated. These kinds of experiments are laborious and expensive due to difficulties in obtaining infective piroplasm isolates, the raising of laboratory colonies of ticks of appropriate species (including artificial feeding and infection of ticks), and/or access to specific vertebrate hosts of babesiae. Therefore, it is not surprising that the great majority of studies on species of *Babesia* in ticks are presently based on field-derived data, with the application of molecular techniques for the detection of DNA of the piroplasm in questing and/or engorged ticks [14, 21–30].

In the case of field-derived data, the detection of *Babesia* DNA in engorged ticks (of any life stage) collected from human or animal hosts is only indicative of the acquisition of piroplasms from an infected host. It is worth remembering that, although the majority of humans are free of tick-borne pathogens, piroplasm infections may be very common among free-living animals (i.e. > 80% in roe deer and > 60% in red foxes; [21]) or circulating among pets and livestock [26, 31]. Whereas detection of *Babesia* DNA in questing (host-seeking) larvae suggests successful transovarial transmission, detection in questing nymphs or adult ticks indicate that babesiae were both acquired during the blood meal in the preceding life stadium and passed through the moult (transstadial transmission) [20, 32], confirming the occurrence of at least two of the key processes mentioned above.

However, field-derived data alone can never satisfy the final criterion of vector competence (the unequivocal demonstration of the transmission of babesiae by a feeding tick), but may provide important information on actual health risks constituted by certain tick species in certain regions, habitats or conditions.

## Confirmed and unconfirmed interactions between *Babesia* and *Ixodes* spp.

### Confirmed interactions between *Babesia capreoli*, *Babesia divergens*, *Babesia microti*, *Babesia venatorum* and *I. ricinus*

*Ixodes ricinus* has been the best-examined tick species for babesiae in recent years [33–104], with the wide application of molecular techniques for piroplasm identification resulting in the confirmation of a specific vector role of this tick species for at least four species of *Babesia*: *B. venatorum*, *B. microti*, *B. divergens* and *B. capreoli* (Additional file 2: Table S1). Interestingly, in the papers published between 2000 and 2010, mostly *B. microti* and *B. divergens* were reported in *I. ricinus*, and only in the last 5–10 years have the range and ranking of *Babesia* species expanded and changed. *Babesia venatorum* (previously known as ‘*Babesia* sp. EU1’) has been more frequently reported in *I. ricinus* since its identification as a species separate from *B. divergens* [105], and seems to be more common/widespread than *B. microti* or *B. divergens* (Additional file 2: Table S1). Similarly, since the detailed re-description of *B. capreoli* by Malandrin et al. [106] in a study which also provided a simple method to differentiate between *B. capreoli* and *B. divergens* based on the presence of three single nucleotide polymorphisms in a complete 18S ribosomal DNA sequence (rDNA), both the recognition and reported prevalence of *B. capreoli* in *I. ricinus* have increased. It is worth underlining here that *B. capreoli*, *B. venatorum* and *B. divergens* all belong to the *Babesia* sensu stricto group (clade X; [107]) and share a high similarity (up to 99.8% identity; [105, 106]) in the conserved 18S rRNA gene. Consequently, before wide recognition of *B. capreoli* and *B. venatorum*, these two species could have been (mis)identified as *B. divergens* or *B. divergens*-like, and this (mis)identification could have contributed to a higher reported prevalence of *B. divergens* in papers published in the period between 2000 and 2010 (Additional file 2: Table S1). It has also contributed to misidentification of *B. divergens* in human cases of babesiosis [105]. Better awareness of this is still needed for differentiation between these three *Babesia* species. Moreover, co-infection of ticks with different combinations of *B. venatorum*, *B. capreoli* and *B. divergens* has also been reported in several recent studies [21], and may have contributed to the lack of proper identification of the species involved. *Ixodes ricinus* ticks can acquire these three *Babesia* species when feeding on domestic and free-living ungulates, including cattle (acquisition of *B. divergens*), roe deer (*Capreolus capreolus*; acquisition of *B. capreoli* and *B. venatorum*) and red deer ((*Cervus elaphus*; acquisition of *B. divergens*) [21, 106, 108–110]. In natural conditions, deer species (roe deer *Capreolus capreolus* and red deer *Cervus elaphus*) are considered the most important sources of a blood meal for *I. ricinus* females, and the presence/density of deer is positively associated with the occurrence/density of *I. ricinus* [111].

Among the numerous studies on *Babesia* in *I. ricinus* ticks, the largest dataset (between 18,000 and 25,000 examined ticks) originated from long-term (2000–2019) studies in the Netherlands and Belgium (Additional file 2: Table S1; [21]). Four *Babesia* species from two clades and a *Babesia* sp. deer genotype were identified in this dataset: *B. venatorum* (210 positive ticks, prevalence 0.8%); *B. microti*-like [45 sequences of *B. microti*, prevalence of *B. microti*-like (clade 1) 2.6%]; *B. capreoli* (11 positive ticks, prevalence 0.04%); *B. divergens* (four positive ticks, prevalence 0.01%); and *Babesia* sp. deer genotype (*Babesia odocoilei*-like, one sequence, prevalence < 0.01%).

Additional evidence supporting the specific interactions between *I. ricinus* and these four *Babesia* species is the repeated observations of these babesiae in different European countries (Additional file 2: Table S1). Interestingly, apart from a single observation for *D. reticulatus*, these species of *Babesia* have not been observed in other (questing) tick species that did not belong to the genus *Ixodes* (Table 1). Three of these species were additionally identified in two other *Ixodes* species from Eurasia, i.e. *B. capreoli*, *B. microti*, and *B. venatorum* in *Ixodes persulcatus* from Mongolia, Russia and Japan, and *B. microti* in *Ixodes pavlovskyi* from Russia (Additional file 2: Table S1; [112]). These tick species constitute the ‘*I. ricinus* complex’, thus the observed *Babesia*-tick interactions may be specific for all the species in the complex; however, this idea needs further investigation.

**Table 1:** Species of *Babesia* reported in *Dermacentor* spp.

Country	Reference	*Dermacentor* species (*n*)	*Babesia* spp. prevalence	Species of *Babesia*, number of isolates, prevalence (%)	Species identification method
Austria	Hodžić et al. [155]	*Dermacentor reticulatus* (128)	10%	*Babesia canis*, 9 (7%)	PCR sequencing
				*Babesia vulpes*, 4 (3%)	
Austria	Leschnik et al. [161]	*D. reticulatus*^a^ (12)	16.7%	*B. canis*, 2 (16.7%)	PCR sequencing
Belgium, the Netherlands, Germany, UK	Sprong et al. [162]	*D. reticulatus* (1741)	0.9%	*B. canis*, 16 (0.9%)	PCR sequencing
Belgium, the Netherlands	Jongejan et al. [139]	*D. reticulatus* (855)	1.9%	*B. canis*, 14 (1.6%)	PCR sequencing
				*Babesia caballi*, 2 (0.2%)	
France	Bonnet et al. [140]	*Dermacentor marginatus* (377)	0.6%	*Babesia bovis*, 1 (0.3%)	PCR-RLB for selected *Babesia* species
				*Babesia/Theileria* spp., 1 (0.3%)	
		*D. reticulatus* (74)	0%	-	
Germany	Galfsky et al. [50]	*D. reticulatus* (30)	3.3%	*Babesia capreoli*, 1 (3.3%)	PCR sequencing
Germany	Silaghi et al. [163]	*D. reticulatus* (301)	0.3%	*B. canis*, 1 (0.3%)	PCR sequencing
Hungary	Hornok et al. [164]	*D. reticulatus* (413)	8.2%	*B. canis*, 34 (8.2%)	PCR sequencing
Lithuania and Latvia	Radzijevskaja et al. [67]	*D. reticulatus* (2440)	1.3%	*B. canis*, 17	PCR sequencing
				*Babesia venatorum*, 1	
Poland	Bajer et al. [131]	*D. reticulatus* (29)	3.4%	*B. canis*, 1 (3.4%)	PCR sequencing
Poland	Mierzejewska et al. [137]	*D. reticulatus* (2585)	4.2% (108)	*B. canis*, 57	PCR sequencing
				*Babesia microti* Munich, 1	
Poland	Wojcik-Fatla et al. [165]	*D. reticulatus* (468)	4.5%	*B. microti* Munich, 21 (4.5%)	PCR sequencing
Poland	Wojcik-Fatla et al. [74]	*D. reticulatus* (582)	2.7%	*B. microti*, 12 (2.1%)	PCR sequencing
				*B. canis*, 4 (0.7%)	
Romania	Corduneanu et al. [166]	*D. reticulatus* (75 in 15 pools)	8% MIR	*B. canis*, 6 (8% MIR)	PCR sequencing
Russia	Rar et al. [167]	*D. reticulatus* (81)	3.6%	*B. canis*, 3 (3.6%)	PCR sequencing
Slovakia	Majláthová et al. [168]	*D. reticulatus* (326)	36%	*B. canis*, 5	PCR sequencing
Slovakia	Svehlová et al. [80]	*D. reticulatus* (600)	1.8%	*B. canis*, 11 (1.8%)	PCR sequencing
Slovenia	Duh et al. [169]	*D. reticulatus* (100)	1%	*B. canis*, 1 (1%)	PCR sequencing
Spain	Garcia-Sanmartin et al. [125]	*D. reticulatus* (97)	5%	*B. canis*, 1 (1%)	PCR-RLB
				*B. caballi*, 1 (1%)	
				*B. caballi*-like, 2 (2%)	
				*Babesia bigemina*, 1 (1%)	
				*Babesia divergens*, 2 (2%)	
Switzerland	Schaarschmidt et al. [88]	*D. reticulatus* (23)	39%	*B. canis*, 9 (39%)	PCR sequencing
Ukraine	Karbowiak et al. [170]	*D. reticulatus* (205)	3.4%	*B. canis*, 4	PCR sequencing
Ukraine	Rogovskyy et al. [90]	*D. reticulatus* (98)	4%	*B. canis*, 1 (1%)	PCR sequencing
				*Babesia odocoilei*-like, 3 (3%)	
USA	Swei et al. [144]	*Dermacentor albipictus* (471 questing larvae)	7.2%	*Babesia duncani* (2 strains: WA1 And BH3), 34 (7.2%)	PCR sequencing
China	Abdallah et al. [171]	*Dermacentor silvarum* (84)	4.8%	*Babesia motasi*-like, 3 (3.6%)	RLB, PCR sequencing
				*Babesia* sp. Xinjiang, 1 (1.2%)	
Mongolia	Battsetseg et al. [153]	*Dermacentor nuttalli* (108 = 54 pools)	6.5% MIR	*B. caballi*, 7 (6.5% MIR)	Species-specific PCR

*MIR* Minimal infection rate, *PCR* polymerase chain reaction, *RLB* reverse line blot

^a^Questing and feeding ticks

**Fig. 1 a–j:**
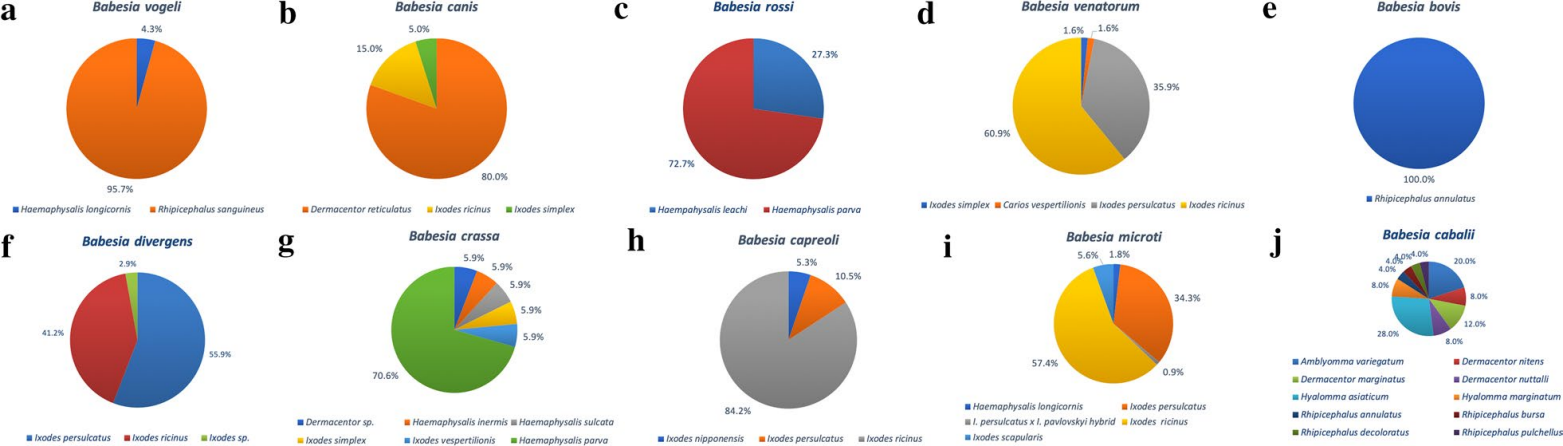
Percentage share of certain tick species as the source of 18S ribosomal DNA (rDNA) sequences of specific *Babesia* species. **a** 70 sequences of *Babesia vogeli*: from Brazil (n = 1), China (n = 5), Cuba (n = 1), Egypt (n = 4), France (n = 29), India (n = 4), Portugal (n = 1), Taiwan (n = 22), Tunisia (n = 1), Palestine (n = 2). **b** 41 sequences of *Babesia canis*: from Austria (n = 1), Hungary (n = 5), Italy (n = 2), Kazakhstan (n = 1), Latvia (n = 1), Lithuania (n = 6), Poland (n = 6), Romania (n = 3), Russia (n = 2), Serbia (n = 2), Slovakia (n = 7), Ukraine (n = 4), UK (n = 1). **c** 11 sequences of *Babesia rossi*: from Nigeria (n = 3), Turkey (n = 8). **d** 64 sequences of *Babesia venatorum*: from China (n = 3), Czech Republic (n = 4), Germany (n = 2), Japan (n = 1), Latvia (n = 6), Lithuania (n = 2), Mongolia (n = 14), Norway (n = 12), Romania (n = 1), Russia (n = 2), Slovakia (n = 1), Sweden (n = 15), Great Britain (n = 1). **e** *Babesia bovis*: four sequences from Egypt. **f** 34 sequences of *Babesia divergens*: from Belgium (n = 3), China (n = 5), Germany (n = 3), Japan (n = 14), Luxembourg (n = 1), the Netherlands (n = 1), Norway (n = 3), Russia (n = 1), Sweden (n = 1), Switzerland (n = 2). **g** 17 sequences of *Babesia crassa*: from China (n = 1), Hungary (n = 3), Russia (n = 1), Turkey (n = 12). **h** 19 sequences of *Babesia capreoli*: from Belgium (n = 2), Germany (n = 6), Latvia (n = 2), Italy (n = 2), Norway (n = 2), Poland (n = 2), Slovakia (n = 2), South Korea (n = 1). **i** 102 sequences of *Babesia microti*: from Austria (n = 1), Belarus (n = 3), Belgium (n = 3), China (n = 2), Estonia (n = 8), Germany (n = 27), Japan (n = 4), Latvia (n = 11), Lithuania (n = 1), Luxembourg (n = 3), Mongolia (n = 21), Poland (n = 2), Russia (n = 3), Slovakia (n = 1), Sweden (n = 10), Ukraine (n = 2), USA (n = 6). **j** 25 sequences of *Babesia caballi*: from Brazil (n = 2), Bulgaria (n = 1), China (n = 7), Ethiopia (n = 1), Guinea (n = 2), Italy (n = 1), Kenya (n = 4), Malaysia (n = 3), Mongolia (n = 2)

More evidence for the specificity of the interactions between these four *Babesia* species and ticks from the *I. ricinus* complex was obtained from data deposited in GenBank. The data are presented in Fig. 1 as percentage share of each tick species from which certain *Babesia* sequences were obtained. Clearly, *I. ricinus* and *I. persulcatus* are the main sources of numerous *B. venatorum*, *B. divergens* and *B. capreoli* sequences (95–97% of all deposited 18S rDNA sequences), and are significant sources of *B. microti* sequences.

*Babesia microti* is one of these four species commonly reported in *I. ricinus* (Additional file 2: Table S1). In many of the studies conducted at the beginning of the present century this piroplasm species was reportedly the most common one in *I. ricinus* ticks in Europe, although again, some of the results may be misleading as PCR products were not sequenced in any of these studies, and all positive PCR results were assumed to indicate *B. microti* infections. There is also a high discrepancy between the reported prevalences of *B. microti* in ticks (Additional file 2: Table S1). Rodents constitute the main reservoir hosts and the main source of *B. microti* infection for *I. ricinus* ticks [113–116], especially for larvae and nymphs which feed on rodents in woodland and open habitats [23, 117, 118].

Interestingly, although more species of ticks feed as juveniles on rodents, *B. microti* has been rarely reported in tick species other than *I. ricinus*, although again, *B. microti* DNA has been repeatedly identified in engorged ticks of different species (*Ixodes trianguliceps*, *D. reticulatus*, *Haemaphysalis concinna* [23, 28, 30]. Interestingly, both main *B. microti* strains, of which one is potentially zoonotic (US type, Jena) and the other non-zoonotic (Munich), were identified in *I. ricinus* ticks from different European countries and at different frequencies [30, 112, 114, 119].

*Babesia microti* has also been reported in other species of the *I. ricinus* complex, as mentioned previously (Additional file 2: Table S1; Fig. 1). *Babesia microti* (US type, Hobetsu, Kobe) has also been found in ticks in Japan, with a zoonotic US type identified in *I. persulcatus* ticks [120]. However, the most significant characteristic of this piroplasm is the role of *I. scapularis* as its vector in the USA, where this *Babesia* species is responsible for the majority of human cases, including fatal and congenital cases [121], and one of the reasons that Yang et al. [7] declared this region ‘Ground Zero’ for human babesiosis. The majority of tick studies in the USA have been focused on *I. scapularis* for this reason, and *Babesia* cf*. microti* has been found additionally, to date, only in one study, in two questing *Amblyomma americanum* ticks (Table 2). Thus, the specificity of the *B. microti-I. scapularis* interaction based on environmental studies in the USA is well documented (Additional file 2: Table S1) and the relevant sequences have been deposited in the GenBank database (Fig. 1i).

**Table 2:** Species of *Babesia* reported in tick species other than *Ixodes* or *Dermacentor* spp.

Country	Reference	Tick species (*n*)	*Babesia* spp. prevalence	*Babesia* species, number of isolates and prevalence (%)	Species identification method
Czech Republic, Slovakia	Rybarova et al. [126]	*Haemaphysalis concinna* (150)	4%	*Babesia* sp., 6 (4%)	PCR sequencing
USA	Shock et al. [172]a	*Amblyomma americanum* (184, including questing)	3.3%	*Babesia* cf. *microti*, 2 (from questing)	PCR sequencing
China	Abdallah et al. [171]	*Haemaphysalis qinghaiensis* (242)	13.5%	*Babesia* sp. Xinjiang, 32 (13.2%)	PCR-RLB, PCR sequencing
				*Babesia bovis*, 1 (0.4%)	
China	Li et al. [173]	*Rhipicephalus microplus* (459)	0.4%	*Babesia bigemina*, 2 (0.4%)	PCR sequencing
China	Zhuang et al. [174]	*Haemaphysalis longicornis* (144)	0.7%	*Babesia* sp., 1 (0.7%)	NGS
China	Niu et al. [175]	*Haemaphysalis qinghaiensis* (188)	21.3%	*Babesia* sp. Xinjiang, 40 (21.3%)	Species-specific PCR
		*Haemaphysalis longicornis* (113)	9.7%	*Babesia* sp. Xinjiang, 11 (9.7%)	
Hungary	Hornok et al. [150]	*Haemaphysalis inermis* (315)	NC	*Babesia crassa*-like, ten pools	PCR sequencing (pools)
		*Haemaphysalis concinna* (259)	NC	*Babesia* sp. Kh-Hc222, one pool	
				*Babesia* sp. Irk-Hc133, four pools	
		*Haemaphysalis punctata* (61)	NC	No *Babesia*	
Spain	Garcia-Sanmartin et al. [125]	*Haemaphysalis inermis* (87)	1.1%	*B. bigemina*, 1 (1.1%)	PCR-RLB
		*Haemaphysalis punctata* (111)	4.5%	*B. bigemina*, 1 (0.9%)	
				*B. bovis*, 1 (0.9%)	
				*Babesia caballi*, 1 (0.9%)	
				*B. caballi*-like, 1 (0.9%)	
				*Babesia vulpes* (*Theileria annae*), 1 (0.9%)	
		*Haemaphysalis concinna* (24)	0%	*-*	
		*Rhipicephalus bursa* (50)	4%	*B. caballi*, 1 (2%)	
				*Babesia ovis*, 1 (2%)	
Slovakia	Hamšíková et al. [116]	*Haemaphysalis concinna* (91)	6.6%	*Babesia* sp. 1 (Eurasia), 5	PCR sequencing
				*Babesia* sp. 2 (Eurasia), 1	
Turkey	Brinkmann et al. [176]	*Rhipicephalus bursa* (76)	1.3%	*B. ovis*, 1 (1.3%)	NGS
Turkey	Orkun et al. [151]	*Haemaphysalis parva* (793)	1.6%	*B. crassa*, n = 8 (1%)	PCR sequencing
				*Babesia rossi*, 4 (0.5%)	
				*Babesia* sp., 1 (0.1%)	
		*Hyalomma marginatum* (105)	12%	*Babesia occultans*, 12 (11%)	
				*Babesia* sp. tavsan 1 1 (1%)	
		*Rhipicephalus turanicus* (9)	11%	*Babesia* sp. tavsan 2 1 (11%)	
Israel	Harrus et al. [177]	*Rhipicephalus turanicus* (83 pools)	1.2% MIR	*Babesia vogeli*, one pool (1.2%)	PCR sequencing (pools)
		*Rhipicephalus sanguineus* (48 pools)	4.2% MIR	*B. vogeli*, two pools (4.2%)	
		*Hyalomma* spp. (13 specimens)	0%	*-*	
Italy	Romiti et al. [152]	*Rhipicephalus bursa* (980 in 110 pools)	14.6% pools	*B. caballi*, 16 pools (14.5%)	qPCR with TaqMan probe for *B. caballi*
Japan	Masatani et al. [178]^a^	*Haemaphysalis formosensis* (159)	1.3%	*Babesia* sp. (feral raccoon strain) (1.3%)	PCR sequencing
		*Haemophysalis flava* (191)	1.6%	*Babesia* sp. (feral raccoon strain) (1.6%)	
		*Haemophysalis longicornis* (219)	0%	*-*	
Japan	Sivakumar et al. [101]	*Haemophysalis longicornis* (175)	9.7%	*Babesia ovata*, 17 (9.7%)	Species-specific PCR for *B. ovata*
Thailand	Wattanamethanont et al. [179]	*Haemaphysalis lagrangei* (11,309), *Haemaphysalis wellingtoni* (16), *Rhipicephalus microplus* (859); total of 419 tick pools	0.2% (1/419 pools)	*Babesia* sp. (new), 1 (0.2% pools)	PCR sequencing

MIR for tick pools

*NGS* Next-generation sequencing,* qPCR* quantitative PCR, *NC* not calculated (pools with different number of ticks tested); for other abbreviations, see Table 1

^a^Mostly questing, but also some feeding ticks tested together

### Confirmed interactions between *B. odocoilei* and *I. scapularis *and between *B. odocoilei*-like and *I. ricinus*

In contrast to *I. ricinus*, in *I. scapularis* only one other *Babesia* species has been identified, *B. odocoilei* in ticks from Canada and the USA (Additional file 2: Table S1). In Canada, *B. odocoilei* was found to be the prevailing species [122–124]. This is another babesiae with deer as its main vertebrate host (American white-tailed deer, *Odocoileus virginianus*) [124]. Interestingly, also in Europe, DNA of a *Babesia* sp. genetically similar to *B. odocoilei* (*B. odocoilei*-like or ‘deer genotype’) was detected several times in *I. ricinus* ticks (Additional file 2: Table S1; [21, 108]). However, this interaction needs more studies to support its relevance. In summary, molecular data from 20 years of eco-epidemiological studies support the role of *I. ricinus* (or *I. ricinus* complex) as a vector of two babesiae clades, I and X [107], associated with two groups of reservoir hosts, deer and rodents.

### Unconfirmed interactions between *Babesia bigemina*, *Babesia bovis*, *Babesia caballi*, *B. caballi*-like, *Babesia canis*, *Babesia major*, *Babesia ovis*, *Babesia vulpes* and *I. ricinus*

The available molecular studies on questing *I. ricinus* ticks do not support interactions between *B. bigemina*, *B. bovis*, *B. caballi*, *B. caballi*-like, *B. canis*, *B. major*, *B. ovis* or *B. vulpes* and *I. ricinus*. Also, the available sequences of these *Babesia* species do not support the role of *I. ricinus* as their vector (Fig. 1). The majority of these *Babesia* species have been reported only in one study, which used a PCR-reverse line blot (RLB) method [125]. Considering the high number of studies on these *Babesia* species, together with the wide range of diagnostic methods applied (PCR sequencing, nested PCR, quantitative PCR, next-generation sequencing), it is highly probable that *I. ricinus* ticks are not vectors for them. The highest number of these studies concern *B. canis*, which was reported from the Czech Republic and Poland [126–128]. However, the authors of the first study, Rybarova et al. [126], concluded that *B. canis* may have been misidentified, possibly as a consequence of the short-sequence PCR product, and thus requires further investigation [126]. In Poland, a recent analysis of the distribution of *D. reticulatus* and outbreaks of canine babesiosis found strong geographical and temporal (seasonal) associations between them [129], which would be less likely if *I. ricinus* were also a competent vector of this piroplasm.

## Interactions between *Babesia* and *Dermacentor* spp.

### Confirmed interaction between *B. canis* and *D. reticulatus*

The ornate dog tick is both the second most common tick species in Europe and the second-best studied tick species (Table 1). Other *Dermacentor* species have been much less studied. Although a range of babesiae have been reported in *D. reticulatus*, the most common and widespread one is *B. canis* (Table 1), the main cause of canine babesiosis in central and north-eastern Europe [130–133]. The great majority (> 80%) of *B. canis* sequences originate from the tick species *D. reticulatus* (Fig. 1). Interestingly, the geographical range of this tick species is expanding in many European countries [129, 134, 135], and this expansion is clearly associated with the emergence of canine babesiosis, although in some tick populations DNA of *B. canis* has not yet been found [136, 137]. During our long-term studies (since 2012 up until the present) on the expansion of the distributions of *D. reticulatus* and *B. canis* in Poland, we have examined the highest number of questing adult ticks for *Babesia* spp. to date (Additional file 2: Table S1; [137]). About 100 *Babesia* sequences were derived from at least 200 *Babesia*-positive ticks, all but one identified as *B. canis* [32, 132, 137]. In addition, DNA of *B. microti* was identified in one adult *D. reticulatus* tick [137]. Interestingly, the opposite occurrence of these two *Babesia* species was found in juvenile, partially engorged *D. reticulatus* ticks (larvae and nymphs) collected from rodents, where *B. microti* constituted the majority of *Babesia*-positive samples, and only two samples yielded *B. canis* DNA [23]. As larvae and nymphs of *D. reticulatus* feed on rodents, and mainly on voles (*Microtus* and *Alexandromys* spp.), the key reservoir of *B. microti* (over 60% of voles infected in three studies [21, 30, 114]), the detection of *B. microti* DNA in engorged instars collected directly from these hosts is not surprising. More surprising is the apparent loss of *B. microti* during the moult of instars to the adult stadium, as DNA of *B. microti* is sporadically found in questing adult *D. reticulatus* ticks (Table 1). Transovarial and transstadial transmissions of *B. canis* in *D. reticulatus* ticks constitute the key routes enabling maintenance of this piroplasm in tick populations [32] and are in contrast with unsuccessful transstadial transmission of *B. microti* in this tick species, as can be seen in the results of the eco-epidemiological studies listed in Table 1. Thus it is highly unlikely that *D. reticulatus* plays any role as a vector of *B. microti*, and the identification of DNA of *B. microti* in adult ticks can be the result of the detection of blood remnants of previous stages that have fed on infected rodents [138].

The possible role of *D. reticulatus* as a vector of *B. caballi* (aetiological agent of equine babesiosis) seems questionable in light of the numerous studies (Table 1), as DNA of *B. caballi* was detected only once, in two questing ticks in the Netherlands [139]. The second report on *B. caballi* in *D. reticulatus* was based on PCR-RLB method [125]. In that study, many other *Babesia* spp. were found in *D. reticulatus* ticks (Tables 1, 2; Additional file 2: Table S1). However, as there is little or no support from other field studies for these findings, the role of *D. reticulatus* as a vector of *B. bigemina* or *B. divergens* is considered doubtful (Table 1).

### Unconfirmed interactions between *B. bigemina*, *B. caballi*, *B. capreoli*, *B. divergens*, *B. microti*, *B. odocoilei*-like, *B. venatorum*, *B. vulpes* and *D. reticulatus*

Despite the high number of studies carried out in large geographical areas, there are only a few reports of *B. bigemina*, *B. caballi*, *B. capreoli*, *B. divergens*, *B. microti*, *B. odocoilei*-like, *B. venatorum* or *B. vulpes* in *D. reticulatus* (Table 1). Thus the role of this tick species as their vector is not supported by published eco-epidemiological studies.

### *Babesia bovis*-*Dermacentor marginatus* interaction

The only available field study, from France [140], on questing *D. marginatus* ticks reported one tick infected with *B. bovis* (Table 1). More studies are needed on field-collected ticks from different areas where *D. marginatus* occurs.

### *Babesia duncani*-*Dermacentor albipictus* interaction

*Babesia duncani* is a quite recently described species, and causes human babesiosis in western USA [141, 142]. *Babesia duncani* was first isolated in 1991 from a patient from Washington State, USA, and was then referred to as ‘*Babesia* strain WA1’ [143]. To date, there have been 12 confirmed human cases of babesiosis due to *B. duncani*, two presumed cases that preceded the description of *B. duncani* in the USA [144], and a rapidly increasing number of suspected cases in Canada [7]. *Babesia duncani* has not been found in questing *I. scapularis* (Additional file 2: Table S1). Swei et al. [144] provide evidence from their recent field study that the vector for *B. duncani* is the winter tick *D. albipictus* (Table 1), and the reservoir host is likely the mule deer *Odocoileus hemionus*. Interestingly, broad, overlapping ranges of these two species cover a large portion of far-western North America, where the human cases were identified. Swei et al.’s [144] study was focused on the detection of *Babesia* DNA in questing ticks, so the authors attempted to collect the only questing stadium in the life cycle of *D. albipictus*, larvae, and were able to support their research hypothesis by the detection of DNA of *B. duncani* in 7% of field-collected larvae. However, to further support this hypothesis, more field studies are needed.

## Interactions between *Babesia* and *Haemaphysalis* spp.

### Confirmed interactions between *B. crassa*-like and *Haemaphysalis concinna* and between *B. crassa* and *Haemaphysalis parva*

**Fig. 2:**
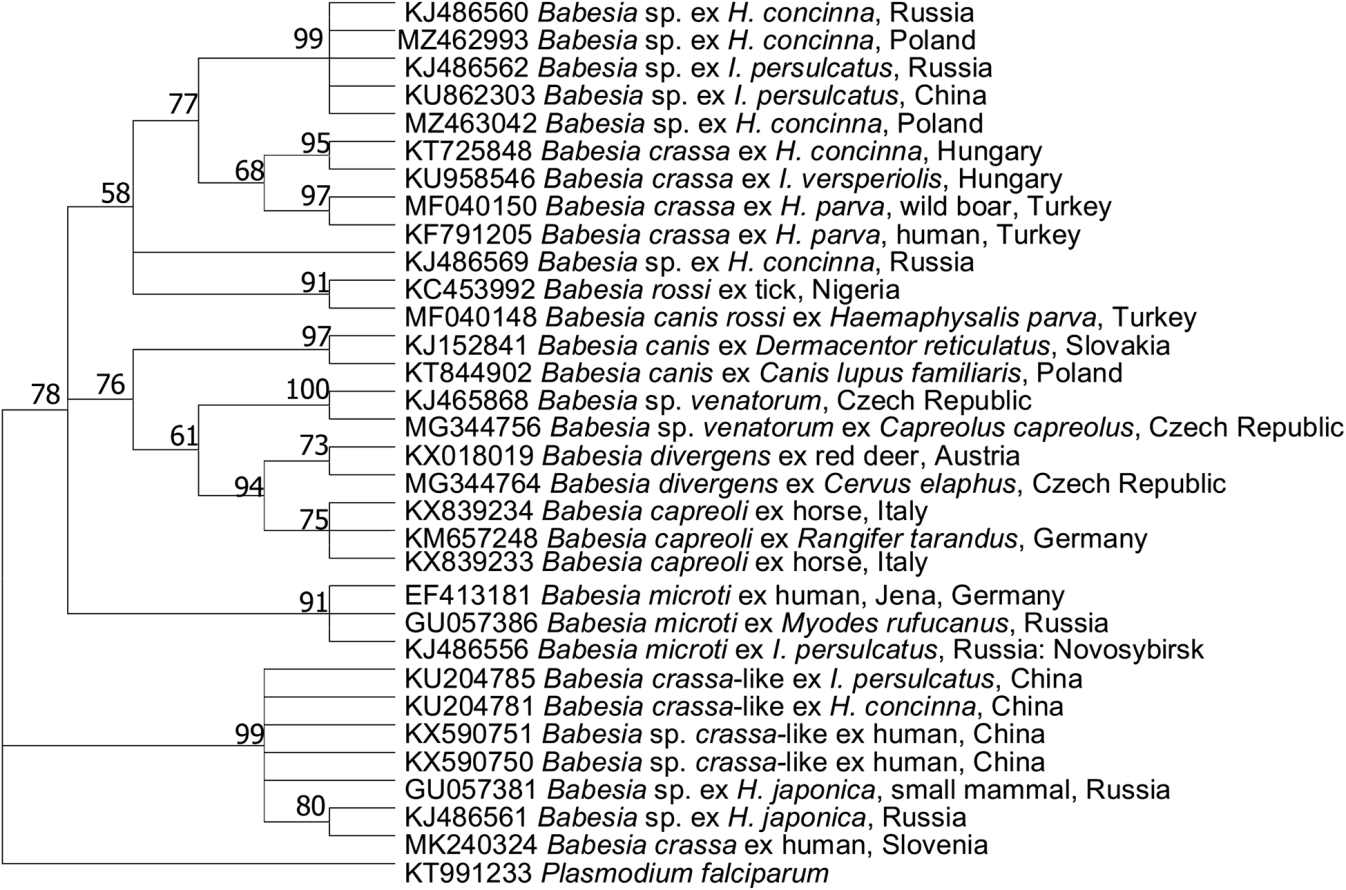
Molecular phylogenetic analysis of 18S rDNA of selected *Babesia* spp. (550 base pairs). The evolutionary history was inferred by using the maximum likelihood method and the Kimura two-parameter model. The tree with the highest log likelihood (− 2752,03) is shown. The percentage of trees in which the associated taxa clustered together is shown next to the branches. Initial tree(s) for the heuristic search were obtained automatically by applying neighbour-joining and BioNJ algorithms to a matrix of pairwise distances estimated using the maximum composite likelihood approach, and then selecting the topology with a superior log likelihood value. A discrete γ distribution was used to model evolutionary rate differences among sites [five categories (+ G, parameter = 2,1600)]. This analysis involved 32 nucleotide sequences. There were a total of 458 positions in the final dataset. Evolutionary analyses were conducted in MEGA X

The relict tick *H. concinna* occurs in Europe and Asia in isolated, geographically limited locations [145]. Together with *I. ricinus* and *D. reticulatus*, *H. concinna* constitutes an important element of the ectoparasite community of domestic and wild animals and humans in Europe [145–147]. Although there is a rather limited number of studies on *Babesia* in *H. concinna* (Table 2), they encompass a wide geographical area, from central Europe to the Far East. Recent studies have revealed (i) a great diversity of *Babesia* in *H. concinna*; (ii) the presence of unique strains or species of *Babesia*, which could not be identified to species level; (iii) the wide distribution of these strains/species in the world (Table 2); and (iv) the possible role of strains/species with an increasing distribution in human babesiosis, i.e. in China [9]. We recently detected one of these strain/species in two juvenile *H. concinna* ticks collected from rodents in western Poland [148]. Two *Babesia* sequences displayed the highest similarity (97.4 and 100%) to an undescribed *Babesia* species from *H. concinna* in Russia (KJ486560). In a phylogenetic analysis using information on *Babesia* from *H. concinna* available from GenBank (Fig. 2), these two sequences grouped with a few *Babesia* sequences from *I. persulcatus* and *H. concinna* from Russia and China [Fig. 2; [149]; shown in Table 2 for *Babesia* from Hungary [150]). Interestingly, this group of sequences was the most similar (sister group) to those of the ovine piroplasm *B. crassa* (95.7% similarity). The third branch of the tree includes *B. crassa*-like sequences from both human clinical cases [9] and *H. concinna* ticks. According to this phylogenetic tree, at least three different species/strains of *Babesia* are associated with *H. concinna*, and are of some pathogenic potential, thus there is an urgent need for better descriptions and characterizations of babesiae from *H. concinna*.

Interestingly, the majority (71%) of sequences of ovine piroplasm *B. crassa* deposited in GenBank originated from *H. parva*, a well-established vector of this species [1], with some share of other *Haemaphysalis* and *Ixodes* spp. (Fig. 1). This interaction was also reported in a recent study from Turkey ([151]; Table 2). This pattern suggests that, although *H. parva* is a vector of *B. crassa*, *H. concinna* is a vector of *B. crassa*-like species, a likely parasite of free-living ungulates [149]. Additionally, *B. crassa*-like was also identified in one *Haemaphysalis inermis* from Hungary [150].

### Interactions between *Babesia* sp. Xinjiang and* Haemaphysalis qinghaiensis* or *Haemaphysalis longicornis*

In two recent studies from China, new zoonotic *Babesia* sp. Xinjiang was found in 13% of *H. qinghaiensis* (Table 2). The prevalence was also similar in *H. longicornis*, so it is likely that these two *Haemaphysalis* spp. can act as vectors for this species, although more field studies are needed to confirm these interactions.

## Interactions between *Babesia* and other tick species

As can be seen in Table 2, there are only a few studies on *Babesia* in other tick species (questing ticks) despite the availability of suitable molecular techniques (reviewed in [22]). This is partially due to the difficulty of obtaining questing individuals of tick species with life cycles that involve one or two host species, like *Rhipicephalus microplus* or *Hyalomma* spp. Studies on the genera *Rhipicephalus* and *Hyalomma* are mainly focused on feeding ticks, and thus do not provide strong evidence on their role as vectors.

## Confirmed and unconfirmed interactions between *Babesia* and other tick species based on GenBank data

The majority of molecular data (18S rDNA) derived from GenBank confirmed the expectations that arose from earlier experimental studies and field observations (summarized in [1]), and reflect specificity in *Babesia*-tick vector interactions. In the case of *Babesia vogeli*, the majority (96%) of sequences originated from *R. sanguineus* s.l. (Fig. 1a); both *H. parva* (73%) and *Haemaphysalis leachi* (27%) constituted the source of *B. rossi* (Fig. 1b), and *B. canis* originated mostly from *D. reticulatus* (Fig. 1c), as mentioned previously. Sequences of *B. bovis* were derived only from *R. annulatus* (Fig. 1e).

However, in the case of *B. caballi*, with ten tick species assigned to deposited 18S rDNA sequences of this species, there is no evidence of any established interaction (Fig. 1j). Of these ten species, four are *Rhipicephalus* species, three *Dermacentor* spp. (but not *D. reticulatus*), two *Hyalomma* spp. and one *Amblyomma*. In two recent studies, *B. caballi* was found in 16 pools of *R. bursa* in Italy (Table 2; [152]) and in seven *D. nuttalli* from Mongolia [153]. Such a variety of tick species might reflect the ability of *B. caballi* to adapt to transmission in parts of the world where horses are bred and/or our inability to determine the main vector for this *Babesia* species. These days, because anti-tick treatments (acaricides, vaccines [3]) can easily be applied to animals of economic significance (horses, cattle, sheep), *Babesia* species specific for these hosts may have been partially eliminated and thus hard to find in their vectors.

A similar problem concerning the determination of tick vectors exists for the recently described *B. vulpes*, a common parasite of red foxes (*Vulpes vulpes*) in Europe [154]. There are not many sequences of *B. vulpes* derived from ticks in GenBank, although at least six tick species, *I. ricinus*, *Ixodes canisuga*, *Ixodes hexagonus*, *Ixodes kaiseri*, *D. reticulatus* and *H. punctata*, have been identified as vectors of this species [29, 125]. *Babesia vulpes* was found in one study in four *D. reticulatus* in Austria [155], and in another study in one *I. ricinus* and one *H. punctata* in Spain [125]. As can be seen from the data discussed here, there is little evidence from eco-epidemiological studies that *I. ricinus*, *D. reticulatus* or *H. concinna* constitute the main vector of *B. vulpes*. The apparent scarcity of data from the most common tick species, together with one of the highest prevalences of this species of *Babesia* in foxes (30–60%), suggests that nidicolous tick species associated with red foxes, such as *I. hexagonus* or *I. canisuga* [29, 156], are its main vectors. Interestingly, dogs are sporadically found infested with *I. hexagonus* [157], and a few cases of babesiosis due to *B. vulpes* have been also recorded in dogs [5, 154]. Due to their nidicolous habit, it would be problematic to collect unfed ticks of these species and either confirm or exclude their role as vectors of *B. vulpes*.

## New *Babesia* species and their vectors

Few studies have been carried out on tick species other than the three most studied ones (Table 2). However, these studies often reveal new *Babesia* species or strains, e.g. in studies carried out in Turkey, Japan and Thailand (Table 2). These interesting findings should encourage researchers to continue, and expand on, such studies to increase the number of new species described. More records of new *Babesia* species/strains in association with certain tick species are needed to recognize new *Babesia*-vector interactions.

## Detection of *Babesia* spp. in ticks from hosts

There are numerous studies reporting *Babesia* spp. in ticks collected from their hosts, especially ticks collected from dogs, cattle, animals that are hunted (i.e. deer or foxes), birds or small mammals [23, 25, 26, 29, 31, 158]. As mentioned at the beginning of this review, and also in many other reviews [19], the results of such studies can be inconclusive or misleading if no control of host infection is performed at the time of tick collection. When ticks are collected from species of rodents in which *Babesia* infections are common [114, 159, 160], these ticks, regardless of the species, may contain pathogen DNA (‘meal contamination’ [23]). The detection of DNA of certain *Babesia* sp. in engorged/partially engorged ticks should be treated with caution and considered in the light of a possible reservoir role of the vertebrate host for the *Babesia* species in question. As mentioned above, the detection of *B. microti* in a high percentage of *D. reticulatus* larvae feeding on voles does not actually support the role of this tick as a vector of *B. microti* because the parasite is apparently lost during the moult of the tick. Similarly, the detection of any *Babesia* species known to be associated with dogs in ticks collected from dogs (i.e. *B. canis* in *I. ricinus*) should be treated as an accidental finding, not as the discovery of a new *Babesia*—tick vector interaction. Regarding *B. vulpes*, DNA of this piroplasm has been identified in three tick species (*I. ricinus*, *I. hexagonus*, *I. canisuga*) collected from foxes, while the prevalence of *B. vulpes* in foxes was close to 50% [29]. Determination of the presence of a pathogen in a tick collected from a certain host may provide very useful information; however, this information should not be used as proof that the tick in question is a vector of that particular pathogen.

## Conclusions

The application of molecular methods in eco-epidemiological studies may help researchers to identify specific interactions between certain *Babesia* and tick species. Well-supported data for the most common *Babesia* and tick species, i.e. *I. ricinus*, *I. scapularis*, *I. persulcatus* and *D. reticulatus*, have been reported during the past 20 years. Published findings on *Babesia*-tick associations have provided evidence for specific interactions, and also complemented experimental transmission studies because they reflect the actual epidemiological situation in certain habitats, e.g. the actual health hazard constituted by certain *Babesia* and tick species in certain locations. It is worth underlining the importance of the correct choice of methods for studies on *Babesia*-tick interactions. These methods should enable both the detection and accurate identification of a wide range of *Babesia* species in ticks. There are presently many methods/techniques that can be used to perform such studies [22]. The wide use of combined PCR and sequencing methods has enabled the identification/confirmation of new or lesser known species of *Babesia*, such as *B. venatorum* and *B. capreoli*, in the widely studied *I. ricinus* tick. The same methods enabled the identification of new strains/species of *Babesia* in less-studied tick species, such as *H. concinna*, *Haemophysalis flava* and *Rhipicephalus turanicus* (Table 2). The massive amount of data collected thus far for the most common tick species should be complemented by more intensive studies on *Babesia* infection in underrepresented tick species.

## Supplementary Information

**Additional file 1: Text S1**. Range of this review.

**Additional file 2: Table S1**. Species of* Babesia* reported in *Ixodes* spp.

## Data Availability

All data generated or analysed during this study are included in this published article and its additional files.

## Abbreviations

NGS: Next-generation sequencing
PCR: Polymerase chain reaction
qPCR: Quantitative polymerase chain reaction
rDNA: Ribosomal DNA
RLB: Reverse line blot

## References

[1] Gray JS, Estrada-Peña A, Zintl A (2019). Vectors of babesiosis. Annu Rev Entomol.

[2] Krause PJ (2019). Human babesiosis. Int J Parasitol.

[3] Bock R, Jackson L, de Vos A, Jorgensen W (2004). Babesiosis of cattle. Parasitology.

[4] Birkenheuer AJ, Buch J, Beall MJ, Braff J, Chandrashekar R (2020). Global distribution of canine *Babesia* species identified by a commercial diagnostic laboratory. Vet Parasitol Reg Stud Rep.

[5] Solano-Gallego L, Sainz Á, Roura X, Estrada-Pena A, Guadalupe M (2016). A review of canine babesiosis: the European perspective. Parasit Vectors.

[6] Penzhorn BL (2020). Don't let sleeping dogs lie: unravelling the identity and taxonomy of *Babesia canis*, *Babesia rossi* and *Babesia vogeli*. Parasit Vectors.

[7] Yang Y, Christie J, Köster L, Du A, Yao C (2021). Emerging human babesiosis with "Ground Zero" in North America. Microorganisms.

[8] Scott JD, Scott CM (2018). Human babesiosis caused by *Babesia duncani* has widespread distribution across Canada. Healthcare (Basel).

[9] Jia N, Zheng YC, Jiang JF, Jiang RR, Jiang BG, Wei R (2018). Human babesiosis caused by a *Babesia crassa*-like pathogen: a case series. Clin Infect Dis.

[10] Zhou X, Li SG, Wang JZ, Huang JL, Zhou HJ, Chen JH, Zhou XN (2014). Emergence of human babesiosis along the border of China with Myanmar: detection by PCR and confirmation by sequencing. Emerg Microbes Infect..

[11] Jiang JF, Zheng YC, Jiang RR, Li H, Huo QB, Jiang BG (2015). Epidemiological, clinical, and laboratory characteristics of 48 cases of “*Babesia venatorum*” infection in China: a descriptive study. Lancet Infect Dis.

[12] Qi C, Zhou D, Liu J, Cheng Z, Zhang L, Wang L (2011). Detection of *Babesia divergens* using molecular methods in anemic patients in Shandong Province. China Parasitol Res.

[13] Jongejan F, Uilenberg G (2004). The global importance of ticks. Parasitology.

[14] Onyiche TE, Răileanu C, Fischer S, Silaghi C (2021). Global distribution of *Babesia* species in questing ticks: a systematic review and meta-analysis based on published literature. Pathogens.

[15] Adl SM, Simpson AGB, Lane CE, Lukeš J, Bass D, Bowser SS (2012). The revised classification of eukaryotes. J Eukaryot Microbiol.

[16] Adl SM, Bass D, Lane CE, Lukeš J, Schoch CL, Smirnov A (2019). Revisions to the classification, nomenclature, and diversity of eukaryotes. J Eukaryot Microbiol.

[17] Zahler M, Schein E, Rinder H, Gothe R (1998). Characteristic genotypes discriminate between *Babesia canis* isolates of differing vector specificity and pathogenicity to dogs. Parasitol Res.

[18] Zahler M, Rinder H, Schein E, Gothe R (2000). Detection of a new pathogenic *Babesia microti*-like species in dogs. Vet Parasitol.

[19] Estrada-Peña A, Cevidanes A, Sprong H, Millán J (2021). Pitfalls in tick and tick-borne pathogens research, some recommendations and a call for data sharing. Pathogens.

[20] Eisen L (2020). Vector competence studies with hard ticks and *Borrelia burgdorferi* sensu lato spirochetes: a review. Ticks Tick Borne Dis..

[21] Azagi T, Jaarsma RI, van Leeuwen AD, Fonville M, Maas M, Franssen FJ (2021). Circulation of *Babesia* species and their exposure to humans through *Ixodes ricinus*. Pathogens.

[22] Martínez-García G, Santamaría-Espinosa RM, Lira-Amaya JJ, Figueroa JV (2021). Challenges in tick-borne pathogen detection: the case for *Babesia* spp. identification in the tick vector. Pathogens.

[23] Dwużnik D, Mierzejewska EJ, Drabik P, Kloch A, Alsarraf M, Behnke JM (2019). The role of juvenile *Dermacentor reticulatus* ticks as vectors of microorganisms and the problem of ‘meal contamination’. Exp Appl Acarol.

[24] Adamska M, Skotarczak B (2017). Molecular detecting of piroplasms in feeding and questing *Ixodes ricinus* ticks. Ann Parasitol.

[25] Maia C, Ferreira A, Nunes M, Vieira ML, Campino L, Cardoso L (2014). Molecular detection of bacterial and parasitic pathogens in hard ticks from Portugal. Ticks Tick Borne Dis.

[26] Reye AL, Arinola OG, Hübschen JM, Muller CP (2012). Pathogen prevalence in ticks collected from the vegetation and livestock in Nigeria. Appl Environ Microbiol.

[27] Reye AL, Stegniy V, Mishaeva NP, Velhin S, Hübschen JM, Ignatyev G (2013). Prevalence of tick-borne pathogens in *Ixodes ricinus* and *Dermacentor reticulatus* ticks from different geographical locations in Belarus. PLoS ONE.

[28] Bown KJ, Lambin X, Telford GR, Ogden NH, Telfer S, Woldehiwet Z (2008). Relative importance of *Ixodes ricinus* and *Ixodes trianguliceps* as vectors for *Anaplasma phagocytophilum* and *Babesia microti* in field vole. Appl Environ Microbiol.

[29] Najm NA, Meyer-Kayser E, Hoffmann L, Herb I, Fensterer V, Pfister K (2014). A molecular survey of *Babesia* spp. and *Theileria* spp. in red foxes (*Vulpes vulpes*) and their ticks from Thuringia, Germany. Ticks Tick Borne Dis..

[30] Welc-Falęciak R, Bajer A, Behnke JM, Siński E (2008). Effects of host diversity and the community composition of hard ticks (Ixodidae) on *Babesia microti* infection. Int J Med Microbiol.

[31] Levytska VA, Mushinsky AB, Zubrikova D, Blanarova L, Długosz E, Vichova B (2021). Detection of pathogens in ixodid ticks collected from animals and vegetation in five regions of Ukraine. Ticks Tick Borne Dis..

[32] Mierzejewska EJ, Dwużnik D, Bajer A (2018). Molecular study of transovarial transmission of *Babesia canis* in the *Dermacentor reticulatus* tick. Ann Agric Environ Med.

[33] Blaschitz M, Narodoslavsky-Gföller M, Kanzler M, Stanek G, Walochnik J (2008). *Babesia* species occurring in Austrian *Ixodes ricinus* ticks. Appl Environ Microbiol.

[34] Lempereur L, Lebrun M, Cuvelier P, Sépult G, Caron Y, Saegerman C (2012). Longitudinal field study on bovine *Babesia* spp. and *Anaplasma phagocytophilum* infections during a grazing season in Belgium. Parasitol Res.

[35] Václavík T, Balážová A, Baláž V, Tkadlec E, Schichor M, Zechmeisterová K (2021). Landscape epidemiology of neglected tick-borne pathogens in central Europe. Transbound Emerg Dis.

[36] Venclíková K, Mendel J, Betášová L, Blažejová H, Jedličková P, Straková P (2016). Neglected tick-borne pathogens in the Czech Republic, 2011–2014. Ticks Tick Borne Dis.

[37] Rudolf I, Golovchenko M, Sikutová S, Rudenko N, Grubhoffer L, Hubálek Z (2005). *Babesia microti* (Piroplasmida: Babesiidae) in nymphal *Ixodes ricinus* (Acari: Ixodidae) in the Czech Republic. Folia Parasitol (Praha).

[38] Klitgaard K, Kjær LJ, Isbrand A, Hansen MF, Bødker R (2019). Multiple infections in questing nymphs and adult female *Ixodes ricinus* ticks collected in a recreational forest in Denmark. Ticks Tick Borne Dis.

[39] Sormunen JJ, Andersson T, Aspi J, Bäck J, Cederberg T, Haavisto N (2020). Monitoring of ticks and tick-borne pathogens through a nationwide research station network in Finland. Ticks Tick Borne Dis..

[40] Sormunen JJ, Klemola T, Hänninen J, Mäkelä S, Vuorinen I, Penttinenet R (2018). The importance of study duration and spatial scale in pathogen detection-evidence from a tick-infested island. Emerg Microbes Infect.

[41] Akl T, Bourgoin G, Souq ML, Appolinaire J, Poirel MT, Gibert P (2019). Detection of tick-borne pathogens in questing *Ixodes ricinus* in the French Pyrenees and first identification of *Rickettsia monacensis* in France. Parasite.

[42] Bonnet S, Michelet L, Moutailler S, Cheval J, Hébert C, Vayssier-Taussat M, Eloit M (2014). Identification of parasitic communities within European ticks using next-generation sequencing. PLoS Negl Trop Dis.

[43] Jouglin M, Perez G, Butet A, Malandrin L, Bastian S (2017). Low prevalence of zoonotic *Babesia* in small mammals and *Ixodes ricinus* in Brittany, France. Vet Parasitol.

[44] Lebert I, Agoulon A, Bastian S, Butet A, Cargnelutti B, Cèbe N (2020). Distribution of ticks, tick-borne pathogens and the associated local environmental factors including small mammals and livestock, in two French agricultural sites: the OSCAR database. Biodivers Data J..

[45] Paul REL, Cote M, Le Naour E, Bonet SI (2016). Environmental factors influencing tick densities over seven years in a French suburban forest. Parasit Vectors.

[46] Reis C, Cote M, Paul RE, Bonnet S (2011). Questing ticks in suburban forest are infected by at least six tick-borne pathogens. Vector Borne Zoonotic Dis.

[47] Lejal E, Moutailler S, Šimo L, Vayssier-Taussat M, Pollet T (2019). Tick-borne pathogen detection in midgut and salivary glands of adult *Ixodes ricinus*. Parasit Vectors.

[48] Franke J, Fritzsch J, Tomaso H, Straube E, Dorn W, Hildebrandt A (2010). Coexistence of pathogens in host-seeking and feeding ticks within a single natural habitat in central Germany. Appl Environ Microbiol.

[49] Franke J, Hildebrandt A, Meier F, Straube E, Dorn W (2011). Prevalence of Lyme disease agents and several emerging pathogens in questing ticks from the German Baltic coast. J Med Entomol.

[50] Galfsky D, Król N, Pfeffer M, Obiegala A (2019). Long-term trends of tick-borne pathogens in regard to small mammal and tick populations from Saxony, Germany. Parasit Vectors.

[51] Hildebrandt A, Franke J, Schmoock G, Pauliks K, Krämer A, Straube E (2011). Diversity and coexistence of tick-borne pathogens in central Germany. J Med Entomol.

[52] Hildebrandt A, Fritzsch J, Franke J, Sachse S, Dorn W, Straube E (2011). Co-circulation of emerging tick-borne pathogens in Middle Germany. Vector Borne Zoonotic Dis.

[53] Overzier E, Pfister K, Thiel C, Herb I, Mahling M, Silaghi C (2013). Diversity of *Babesia* and *Rickettsia* species in questing *Ixodes ricinus*: a longitudinal study in urban, pasture, and natural habitats. Vector Borne Zoonotic Dis.

[54] Overzier E, Pfister K, Herb I, Mahling M, Böck G, Silaghi C (2013). Detection of tick-borne pathogens in roe deer (*Capreolus capreolus*), in questing ticks (*Ixodes ricinus*), and in ticks infesting roe deer in southern Germany. Ticks Tick Borne Dis.

[55] Springer A, Höltershinken M, Lienhart F, Ermel S, Rehage J, Hülskötter K (2020). Emergence and epidemiology of bovine babesiosis due to *Babesia divergens* on a northern German beef production farm. Front Vet Sci.

[56] Schorn S, Pfister K, Reulen H, Mahling M, Silaghi C (2011). Occurrence of *Babesia* spp, *Rickettsia* spp. and B*artonella* spp. in *Ixodes ricinus* in Bavarian public parks, Germany. Parasit Vectors.

[57] Eshoo MW, Crowder CD, Carolan HE, Rounds MA, Ecker DJ, Haag H (2014). Broad-range survey of tick-borne pathogens in southern Germany reveals a high prevalence of *Babesia microti* and a diversity of other tick-borne pathogens. Vector Borne Zoonotic Dis.

[58] Hartelt K, Oehme R, Frank H, Brockmann SO, Hassler D, Kimmig P (2004). Pathogens and symbionts in ticks: prevalence of *Anaplasma phagocytophilum* (*Ehrlichia* sp.), *Wolbachia* sp., *Rickettsia* sp., and *Babesia* sp. in southern Germany. Int J Med Microbiol.

[59] Hildebrandt A, Pauliks K, Sachse S, Straube E (2010). Coexistence of *Borrelia* spp. and *Babesia* spp. in *Ixodes ricinus* ticks in Middle Germany. Vector Borne Zoonotic Dis.

[60] Egyed L, Elő P, Sréter-Lancz Z, Széll Z, Balogh Z, Sréter T (2012). Seasonal activity and tick-borne pathogen infection rates of *Ixodes ricinus* ticks in Hungary. Ticks Tick Borne Dis.

[61] Aureli S, Galuppi R, Ostanello F, Foley JE, Bonoli C, Rejmanek D (2015). Abundance of questing ticks and molecular evidence for pathogens in ticks in three parks of Emilia-Romagna region of northern Italy. Ann Agric Environ Med.

[62] Capelli G, Ravagnan S, Montarsi F, Ciocchetta S, Cazzin S, Porcellato E (2012). Occurrence and identification of risk areas of *Ixodes ricinus*-borne pathogens: a cost-effectiveness analysis in north-eastern Italy. Parasit Vectors.

[63] Cassini R, Bonoli C, Montarsi F, Tessarin C, Marcer F, Galuppi R (2010). Detection of *Babesia* EU1 in *Ixodes ricinus* ticks in northern Italy. Vet Parasitol.

[64] Floris R, Cecco P, Mignozzi K, Boemo B, Cinco M (2009). First detection of *Babesia* EU1 and *Babesia divergens*-like in *Ixodes ricinus* ticks in north-eastern Italy. Parassitologia.

[65] Zanet S, Ferroglio E, Battisti E, Tizzani P. Ecological niche modeling of *Babesia* sp. infection in wildlife experimentally evaluated in questing *Ixodes ricinus*. Geospat Health. 2020;15(1). 10.4081/gh.2020.843 32575961

[66] Capligina V, Berzina I, Bormane A, Salmane I, Vilks K, Kazarina A (2016). Prevalence and phylogenetic analysis of *Babesia* spp. in *Ixodes ricinus* and *Ixodes persulcatus* ticks in Latvia. Exp Appl Acarol.

[67] Radzijevskaja J, Mardosaitė-Busaitienė D, Aleksandravičienė A, Paulauskas A (2018). Investigation of *Babesia* spp. in sympatric populations of *Dermacentor reticulatus* and *Ixodes ricinus* ticks in Lithuania and Latvia. Ticks Tick Borne Dis.

[68] Reye AL, Hübschen JM, Sausy A, Muller CP (2010). Prevalence and seasonality of tick-borne pathogens in questing *Ixodes ricinus* ticks from Luxembourg. Appl Environ Microbiol.

[69] Øines Ø, Radzijevskaja J, Paulauskas A, Rosef O (2012). Prevalence and diversity of *Babesia* spp. in questing *Ixodes ricinus* ticks from Norway. Parasit Vectors.

[70] Pieniazek N, Sawczuk M, Skotarczak B (2006). Molecular identification of *Babesia* parasites isolated from *Ixodes ricinus* ticks collected in northwestern Poland. J Parasitol.

[71] Siński E, Bajer A, Welc R, Pawełczyk A, Ogrzewalska M, Behnke JM (2006). *Babesia microti*: prevalence in wild rodents and *Ixodes ricinus* ticks from the Mazury Lakes District of north-eastern Poland. Int J Med Microbiol.

[72] Sytykiewicz H, Karbowiak G, Hapunik J, Szpechciński A, Supergan-Marwicz M, Goławska S (2012). Molecular evidence of *Anaplasma phagocytophilum* and *Babesia microti* co-infections in *Ixodes ricinus* ticks in central-eastern region of Poland. Ann Agric Environ Med.

[73] Welc-Falęciak R, Bajer A, Paziewska-Harris A, Baumann-Popczyk A, Siński E (2012). Diversity of *Babesia* in *Ixodes ricinus* ticks in Poland. Adv Med Sci.

[74] Wójcik-Fatla A, Zając V, Sawczyn A, Cisak E, Dutkiewicz J (2015). *Babesia* spp. in questing ticks from eastern Poland: prevalence and species diversity. Parasitol Res.

[75] Rar VA, Epikhina TI, Livanova NN, Panov VV (2011). Genetic diversity of *Babesia* in *Ixodes persulcatus* and small mammals from north Ural and west Siberia, Russia. Parasitology.

[76] Movila A, Dubinina HV, Sitnicova N, Bespyatova L, Uspenskaia I, Efremova G (2014). Comparison of tick-borne microorganism communities in *Ixodes* spp. of the *Ixodes ricinus* species complex at distinct geographical regions. Exp Appl Acarol.

[77] Karlsson ME, Andersson MO (2016). *Babesia* species in questing *Ixodes ricinus*, Sweden. Ticks Tick Borne Dis.

[78] Potkonjak A, Gutiérrez R, Savić S, Vračar V, Nachum-Biala Y, Jurišić A (2016). Molecular detection of emerging tick-borne pathogens in Vojvodina, Serbia. Ticks Tick Borne Dis.

[79] Blaňarová L, Stanko M, Miklisová D, Víchová B, Mošanský L, Kraljik J (2016). Presence of Candidatus *Neoehrlichia mikurensis* and *Babesia microti* in rodents and two tick species (*Ixodes ricinus* and *Ixodes trianguliceps*) in Slovakia. Ticks Tick Borne Dis.

[80] Svehlová A, Berthová L, Sallay B, Boldiš V, Sparagano OA, Spitalská E (2014). Sympatric occurrence of *Ixodes ricinus*, *Dermacentor reticulatus* and *Haemaphysalis concinna* ticks and *Rickettsia* and *Babesia* species in Slovakia. Ticks Tick Borne Dis.

[81] Duh D, Petrovec M, Avsic-Zupanc T (2001). Diversity of *Babesia* infecting European sheep ticks (*Ixodes ricinus*). J Clin Microbiol.

[82] Remesar S, Díaz P, Prieto A, García-Dios D, Panadero R, Fernández G (2021). Molecular detection and identification of piroplasms (*Babesia* spp. and *Theileria* spp.) and *Anaplasma phagocytophilum* in questing ticks from northwest Spain. Med Vet Entomol.

[83] Burri C, Dupasquier C, Bastic V, Gern L (2011). Pathogens of emerging tick-borne diseases, *Anaplasma phagocytophilum*, *Rickettsia* spp., and *Babesia* spp., in *Ixodes* ticks collected from rodents at four sites in Switzerland (canton of Bern). Vector Borne Zoonotic Dis.

[84] Casati S, Sager H, Gern L, Piffaretti J (2006). Presence of potentially pathogenic *Babesia* sp. for human in* Ixodes ricinus* in Switzerland. Ann Agric Environ Med.

[85] Gigandet L, Stauffer E, Douet V, Rais O, Moret J, Gern L (2011). Prevalence of three zoonotic *Babesia* species in *Ixodes ricinus* (Linné, 1758) nymphs in a suburban forest in Switzerland. Vector Borne Zoonotic Dis.

[86] Lommano E, Bertaiola L, Dupasquier C, Gern L (2012). Infections and coinfections of questing *Ixodes ricinus* ticks by emerging zoonotic pathogens in western Switzerland. Appl Environ Microbiol.

[87] Oechslin CP, Heutschi D, Lenz N, Tischhauser W, Péter O, Rais O (2017). Prevalence of tick-borne pathogens in questing *Ixodes ricinus* ticks in urban and suburban areas of Switzerland. Parasit Vectors.

[88] Schaarschmidt D, Gilli U, Gottstein B, Marreros N, Kuhnert P, Daeppen JA (2013). Questing *Dermacentor reticulatus* harbouring *Babesia canis* DNA associated with outbreaks of canine babesiosis in the Swiss Midlands. Ticks Tick Borne Dis.

[89] Didyk YM, Blaňárová L, Pogrebnyak S, Akimov I, Peťko B, Víchová B (2017). Emergence of tick-borne pathogens (*Borrelia burgdorferi* sensu lato, *Anaplasma phagocytophilum*, *Ricketsia raoultii* and *Babesia microti*) in the Kyiv urban parks, Ukraine. Ticks Tick Borne Dis..

[90] Rogovskyy A, Batool M, Gillis DC, Holman PJ, Nebogatkin IV, Rogovska YV (2018). Diversity of *Borrelia* spirochetes and other zoonotic agents in ticks from Kyiv, Ukraine. Ticks Tick Borne Dis.

[91] Aliota MT, Dupuis AP, Wilczek MP, Peters RJ, Ostfeld RS, Kramer LD (2014). The prevalence of zoonotic tick-borne pathogens in *Ixodes scapularis* collected in the Hudson Valley, New York State. Vector Borne Zoonotic Dis.

[92] Edwards MJ, Russell JC, Davidson EN, Yanushefski TJ, Fleischman BL, Heist RO (2019). A 4-yr survey of the range of ticks and tick-borne pathogens in the Lehigh Valley region of eastern Pennsylvania. J Med Entomol.

[93] Edwards MJ, Barbalato LA, Makkapati A, Pham KD, Bugbee LM (2015). Relatively low prevalence of *Babesia microti* and *Anaplasma phagocytophilum* in *Ixodes scapularis* ticks collected in the Lehigh Valley region of eastern Pennsylvania. Ticks Tick Borne Dis.

[94] Hersh MH, Ostfeld RS, McHenry DJ, Tibbetts M, Brunner JL, Killilea ME (2014). Co-infection of blacklegged ticks with *Babesia microti* and *Borrelia burgdorferi* is higher than expected and acquired from small mammal hosts. PLoS ONE.

[95] Hutchinson ML, Strohecker MD, Simmons TW, Kyle AD, Helwig MW (2015). Prevalence rates of *Borrelia burgdorferi* (Spirochaetales: Spirochaetaceae), *Anaplasma phagocytophilum* (Rickettsiales: Anaplasmataceae), and *Babesia microti* (Piroplasmida: Babesiidae) in host-seeking *Ixodes scapularis* (Acari: Ixodidae) from Pennsylvania. J Med Entomol.

[96] Milholland MT, Xu G, Rich SM, Machtinger ET, Mullinax JM, Li AY (2021). Pathogen coinfections harbored by adult *Ixodes scapularis* from white-tailed deer compared with questing adults across sites in Maryland, USA. Vector Borne Zoonotic Dis.

[97] Prusinski MA, Kokas JE, Hukey KT, Kogut SJ, Lee J, Backenson PB (2014). Prevalence of *Borrelia burgdorferi* (Spirochaetales: Spirochaetaceae), *Anaplasma phagocytophilum* (Rickettsiales: Anaplasmataceae), and *Babesia microti* (Piroplasmida: Babesiidae) in *Ixodes scapularis* (Acari: Ixodidae) collected from recreational lands in the Hudson Valley Region, New York State. J Med Entomol.

[98] Steiner FE, Pinger RR, Vann CN, Grindle N, Civitello D, Clay K, Fuqua C (2008). Infection and co-infection rates of *Anaplasma phagocytophilum* variants, *Babesia* spp., *Borrelia burgdorferi*, and the rickettsial endosymbiont in *Ixodes scapularis* (Acari: Ixodidae) from sites in Indiana, Maine, Pennsylvania, and Wisconsin. J Med Entomol.

[99] Steiner FE, Pinger RR, Vann CN, Abley MJ, Sullivan B, Grindle N (2006). Detection of *Anaplasma phagocytophilum* and *Babesia odocoilei* DNA in *Ixodes scapularis* (Acari: Ixodidae) collected in Indiana. J Med Entomol.

[100] Scott JD, Pascoe EL, Sajid MS, Foley JE (2021). Detection of *Babesia odocoilei* in *Ixodes scapularis* ticks collected in southern Ontario, Canada. Pathogens.

[101] Sivakumar T, Tattiyapong M, Okubo K, Suganuma K, Hayashida K, Igarashi I (2014). PCR detection of *Babesia ovata* from questing ticks in Japan. Ticks Tick Borne Dis.

[102] Zamoto-Niikura A, Morikawa S, Hanaki KI, Holman PJ, Ishihara C (2016). *Ixodes persulcatus* ticks as vectors for the *Babesia microti* U.S. lineage in Japan. Appl Environ Microbiol.

[103] Karnath C, Obiegala A, Speck S, Essbauer S, Derschum H, Scholz H (2016). Detection of *Babesia venatorum*, *Anaplasma phagocytophilum* and Candidatus *Neoehrlichia mikurensis* in *Ixodes persulcatus* ticks from Mongolia. Ticks Tick Borne Dis.

[104] Tuvshintulga B, Sivakumar T, Battsetseg B, Narantsatsaral SO, Enkhtaivan B, Battur B (2015). The PCR detection and phylogenetic characterization of *Babesia microti* in questing ticks in Mongolia. Parasitol Int.

[105] Herwaldt BL, Cacciò S, Gherlinzoni F, Aspöck H, Slemenda SB, Piccaluga P (2003). Molecular characterization of a non–*Babesia divergens* organism causing zoonotic babesiosis in Europe. Emerg Infect Dis.

[106] Malandrin L, Jouglin M, Sun Y, Brisseau N, Chauvin A (2010). Redescription of *Babesia capreoli* (Enigk and Friedhoff, 1962) from roe deer (*Capreolus capreolus*): isolation, cultivation, host specificity, molecular characterisation and differentiation from *Babesia divergens*. Int J Parasitol.

[107] Jalovecka M, Sojka D, Ascencio M, Schnittger L (2019). *Babesia* life cycle—when phylogeny meets biology. Trends Parasitol.

[108] Fanelli A (2021). A historical review of *Babesia* spp. associated with deer in Europe: *Babesia divergens/Babesia divergens*-like, *Babesia capreoli, Babesia venatorum Babesia *cf.* odocoilei*. Vet Parasitol.

[109] Welc-Falęciak R, Werszko J, Cydzik K, Bajer A, Michalik J, Behnke JM (2013). Co-infection and genetic diversity of tick-borne pathogens in roe deer from Poland. Vector Borne Zoonotic Dis.

[110] Silaghi C, Fröhlich J, Reindl H, Hamel D, Rehbein S (2020). *Anaplasma phagocytophilum* and *Babesia* species of sympatric roe deer (*Capreolus capreolus*), fallow deer (*Dama dama*), sika deer (*Cervus nippon*) and red deer (*Cervus elaphus*) in Germany. Pathogens.

[111] Medlock JM, Hansford KM, Bormane A, Derdakova M, Estrada-Peña A, George JC (2013). Driving forces for changes in geographical distribution of *Ixodes ricinus* ticks in Europe. Parasit Vectors.

[112] Rar V, Livanova N, Tkachev S, Kaverina G, Tikunov A, Sabitova Y (2017). Detection and genetic characterization of a wide range of infectious agents in *Ixodes pavlovskyi* ticks in western Siberia, Russia. Parasit Vectors.

[113] Obiegala A, Pfeffer M, Pfister K, Karnath C, Silaghi C (2015). Molecular examinations of *Babesia microti* in rodents and rodent-attached ticks from urban and sylvatic habitats in Germany. Ticks Tick Borne Dis.

[114] Tołkacz K, Bednarska B, Alsarraf M, Dwużnik D, Grzybek M, Welc-Falęciak R (2017). Prevalence, genetic identity and vertical transmission of *Babesia microti* in three naturally infected species of vole, *Microtus* spp. (Cricetidae). Parasit Vectors.

[115] Silaghi C, Woll D, Hamel D, Pfister K, Mahling M, Pfeffer M (2012). *Babesia* spp. and *Anaplasma phagocytophilum* in questing ticks, ticks parasitizing rodents and the parasitized rodents—analyzing the host-pathogen-vector interface in a metropolitan area. Parasit Vectors.

[116] Hamšíková Z, Kazimírová M, Haruštiaková D, Mahríková L, Slovák M, Berthová L (2016). *Babesia* spp. in ticks and wildlife in different habitat types of Slovakia. Parasit Vectors.

[117] Paziewska A, Zwolińska L, Harris PD, Bajer A, Siński E (2010). Utilisation of rodent species by larvae and nymphs of hard ticks (Ixodidae) in two habitats in NE Poland. Exp App Acarol.

[118] Obiegala A, Arnold L, Pfeffer M, Kiefer M, Kiefer D, Sauter-Louis C (2021). Host-parasite interactions of rodent hosts and ectoparasite communities from different habitats in Germany. Parasit Vectors.

[119] Katargina O, Geller J, Vasilenko V, Kuznetsova T, Järvekülg L, Vene S, Lundkvist Å, Golovljova I (2011). Detection and characterization of *Babesia* species in *Ixodes* ticks in Estonia. Vector Borne Zoonotic Dis.

[120] Zamoto-Niikura A, Tsuji M, Qiang W, Nakao M, Hirata H, Ishihara C (2012). Detection of two zoonotic *Babesia microti* lineages, the Hobetsu and U.S. lineages, in two sympatric tick species, *Ixodes ovatus* and *Ixodes persulcatus*, respectively, in Japan. Appl Environ Microbiol.

[121] Feder HM, Lawlor M, Krause PJ (2003). Babesiosis in pregnancy. N Engl J Med.

[122] Scott JD, Pascoe EL, Sajid MS, Foley JE (2020). Detection of *Babesia odocoilei* in *Ixodes scapularis* ticks collected from songbirds in Ontario and Quebec, Canada. Pathogens.

[123] Scott JD, Clark KL, Coble NM, Ballantyne TR (2019). Detection and transstadial passage of *Babesia* species and *Borrelia burgdorferi* sensu lato in ticks collected from avian and mammalian hosts in Canada. Healthcare (Basel).

[124] Milnes EL, Thornton G, Léveillé AN, Delnatte P, Barta JR, Smith DA (2019). *Babesia odocoilei* and zoonotic pathogens identified from *Ixodes scapularis* ticks in southern Ontario, Canada. Ticks Tick Borne Dis.

[125] Garcia-Sanmartin J, Barandika JF, Juste RA, García-Pérez AL, Hurtado A (2008). Distribution and molecular detection of *Theileria* and *Babesia* in questing ticks from northern Spain. Med Vet Entomol.

[126] Rybarova M, Honsova M, Papousek I, Siroky P (2017). Variability of species of *Babesia* Starcovici, 1893 in three sympatric ticks (*Ixodes ricinus*, *Dermacentor reticulatus* and *Haemaphysalis concinna*) at the edge of Pannonia in the Czech Republic and Slovakia. Folia Parasitol (Praha).

[127] Cieniuch S, Stańczak J, Ruczaj A (2009). The first detection of *Babesia* EU1 and *Babesia canis canis* in *Ixodes ricinus* ticks (Acari, Ixodidae) collected in urban and rural areas in northern Poland. Pol J Microbiol.

[128] Stańczak J, Cieniuch S, Lass A, Biernat B, Racewicz M (2015). Detection and quantification of *Anaplasma phagocytophilum* and *Babesia* spp. in *Ixodes ricinus* ticks from urban and rural environment, northern Poland, by real-time polymerase chain reaction. Exp Appl Acarol.

[129] Dwużnik-Szarek D, Mierzejewska EJ, Rodo A, Goździk K, Behnke-Borowczyk J, Kiewra D (2021). Monitoring the expansion of *Dermacentor reticulatus* and occurrence of canine babesiosis in Poland in 2016–2018. Parasit Vectors.

[130] Adaszek L, Winiarczyk S (2008). Molecular characterization of *Babesia canis canis* isolates from naturally infected dogs in Poland. Vet Parasitol.

[131] Bajer A, Mierzejewska EJ, Rodo A, Bednarska M, Kowalec M, Welc-Falęciak R (2014). The risk of vector-borne infections in sled dogs associated with existing and new endemic areas in Poland. Part 1. A population study on sled dogs during the racing season. Vet Parasitol.

[132] Bajer A, Mierzejewska EJ, Rodo A, Welc-Faleciak R (2014). The risk of vector-borne infections in sled dogs associated with existing and new endemic areas in Poland. Part 2. Occurrence and control of babesiosis in a sled dog kennel during a 13-year-long period. Vet Parasitol.

[133] Földvári G, Široký P, Szekeresm S, Majoros G, Sprong H (2016). *Dermacentor reticulatus*: a vector on the rise. Parasit Vectors.

[134] Rubel F, Brugger K, Pfeffer M, Chitimia-Dobler L, Didyk JM, Leverenz S (2016). Geographical distribution of *Dermacentor marginatus* and *Dermacentor reticulatus* in Europe. Ticks Tick Borne Dis.

[135] Mierzejewska EJ, Estrada-Peña A, Alsarraf M, Kowalec M, Bajer A (2016). Mapping of *Dermacentor reticulatus* expansion in Poland in 2012–2014. Ticks Tick Borne Dis.

[136] Kohn M, Krücken J, McKay-Demeler J, Pachnicke S, Krieger K, von Samson-Himmelstjerna G (2019). *Dermacentor reticulatus* in Berlin/Brandenburg (Germany): activity patterns and associated pathogens. Ticks Tick Borne Dis.

[137] Mierzejewska EJ, Pawełczyk A, Radkowski M, Welc-Falęciak R, Bajer A (2015). Pathogens vectored by the tick, *Dermacentor reticulatus*, in endemic regions and zones of expansion in Poland. Parasit Vectors.

[138] Wodecka B, Skotarczak B (2016). Identification of host blood-meal sources and *Borrelia* in field-collected *Ixodes ricinus* ticks in north-western Poland. Ann Agric Environ Med.

[139] Jongejan F, Ringenier M, Putting M, Berger L, Burgers S, Kortekaas R (2015). Novel foci of *Dermacentor reticulatus* ticks infected with *Babesia canis* and *Babesia caballi* in the Netherlands and in Belgium. Parasit Vectors.

[140] Bonnet S, de la Fuente J, Nicollet P, Liu X, Madani N, Blanchard B (2013). Prevalence of tick-borne pathogens in adult *Dermacentor* spp. ticks from nine collection sites in France. Vector Borne Zoonotic Dis.

[141] Kjemtrup AM, Conrad PA (2000). Human babesiosis: an emerging tick-borne disease. Int J Parasitol.

[142] Conrad PA, Kjemtrup AM, Carreno RA, Thomford J, Wainwright K, Eberhard M (2006). Description of *Babesia duncani* n. sp. (Apicomplexa: Babesiidae) from humans and its differentiation from other piroplasms. Int J Parasitol.

[143] Thomford JW, Conrad PA, Telford SR, Mathiesen D, Bowman BH, Spielman A (1994). Cultivation and phylogenetic characterization of a newly recognized human pathogenic protozoan. J Infect Dis.

[144] Swei A, O'Connor KE, Couper LI, Thekkiniath J, Conrad PA, Padgett KA (2019). Evidence for transmission of the zoonotic apicomplexan parasite *Babesia duncani* by the tick *Dermacentor albipictus*. Int J Parasitol.

[145] Rubel F, Brugger K, Walter M, Vogelgesang JR, Didyk YM, Fu S (2018). Geographical distribution, climate adaptation and vector competence of the Eurasian hard tick *Haemaphysalis concinna*. Ticks Tick Borne Dis.

[146] Dwużnik D, Mierzejewska EJ, Alsarraf M, Bajer A (2019). A new focus of the tick *Haemaphysalis concinna* in western Poland. Exp Appl Acarol.

[147] Duscher GG, Feiler A, Leschnik M, Joachim A (2013). Seasonal and spatial distribution of ixodid tick species feeding on naturally infested dogs from Eastern Austria and the influence of acaricides/repellents on these parameters. Parasit Vectors.

[148] Dwużnik-Szarek D, Mierzejewska EJ, Alsarraf M, Alsarraf M, Bajer A (2021). Pathogens detected in the tick *Haemaphysalis concinna* in western Poland: known and unknown threats. Exp Appl Acarol.

[149] Rar VA, Epikhina TI, Suntsova OV, Kozlova IV, Lisak OV, Pukhovskaya NM (2014). Genetic variability of *Babesia* parasites in *Haemaphysalis* spp. and *Ixodes persulcatus* ticks in the Baikal region and far east of Russia. Infect Genet Evol.

[150] Hornok S, Takács N, Kontschán J, György Z, Micsutka A, Iceton S (2015). Diversity of *Haemaphysalis*-associated piroplasms of ruminants in central-eastern Europe, Hungary. Parasit Vectors.

[151] Orkun O, Çakmak A, Nalbantoğlu S, Karaer Z (2020). Turkey tick news: a molecular investigation into the presence of tick-borne pathogens in host-seeking ticks in Anatolia; initial evidence of putative vectors and pathogens, and footsteps of a secretly rising vector tick,* Haemaphysalis parva*. Ticks Tick Borne Dis..

[152] Romiti F, Magliano A, Antognetti V, Manna G, Cersini A, Scicluna MT (2020). Investigation of ixodid ticks as vectors of *Babesia caballi* and *Theileria equi* (Protozoa: Apicomplexa) in central Italy. J Vector Ecol.

[153] Battsetseg B, Xuan X, Ikadai H, Bautista JL, Byambaa B, Boldbaatar D (2001). Detection of *Babesia caballi* and *Babesia equi* in *Dermacentor nuttalli* adult ticks. Int J Parasitol.

[154] Baneth G, Cardoso L, Brilhante-Simões P, Schnittger L (2019). Establishment of *Babesia vulpes* n. sp. (Apicomplexa: Babesiidae), a piroplasmid species pathogenic for domestic dogs. Parasit Vectors.

[155] Hodžić A, Zörer J, Duscher GG (2017). *Dermacentor reticulatus*, a putative vector of *Babesia* cf *microti* (syn. *Theileria annae*) piroplasm. Parasitol Res.

[156] Dwużnik D, Mierzejewska EJ, Kowalec M, Alsarraf M, Stańczak L, Opalińska P (2020). Ectoparasites of red foxes (*Vulpes vulpes*) with a particular focus on ticks in subcutaneous tissues. Parasitology.

[157] Mierzejewska EJ, Welc-Faleciak R, Karbowiak G, Kowalec M, Behnke JM, Bajer A (2015). Dominance of *Dermacentor reticulatus* over *Ixodes ricinus* (Ixodidae) on livestock, companion animals and wild ruminants in eastern and central Poland. Exp Appl Acarol.

[158] Torina A, Alongi A, Scimeca S, Vicente J, Caracappa S, de la Fuente J (2010). Prevalence of tick-borne pathogens in ticks in Sicily. Transbound Emerg Dis.

[159] Bajer A, Welc-Faleciak R, Bednarska M, Alsarraf M, Behnke-Borowczyk J, Sinski E (2014). Long-term spatiotemporal stability and dynamic changes in the haemoparasite community of bank voles (*Myodes glareolus*) in NE Poland. Microb Ecol.

[160] Tołkacz K, Alsarraf M, Kowalec M, Dwużnik D, Grzybek M, Behnke JM (2018). *Bartonella* infections in three species of *Microtus*: prevalence and genetic diversity, vertical transmission and the effect of concurrent *Babesia microti* infection on its success. Parasit Vectors.

[161] Leschnik MW, Khanakah G, Duscher G, Wille-Piazzai W, Hörweg C, Joachim A (2012). Species, developmental stage and infection with microbial pathogens of engorged ticks removed from dogs and questing ticks. Med Vet Entomol.

[162] Sprong H, Fonville M, Docters van Leeuwen A, Devillers E, Ibañez-Justicia A, Stroo A (2019). Detection of pathogens in *Dermacentor reticulatus* in northwestern Europe: evaluation of a high-throughput array. Heliyon.

[163] Silaghi C, Weis L, Pfister K (2020). *Dermacentor reticulatus* and *Babesia canis* in Bavaria (Germany)—a georeferenced field study with digital habitat characterization. Pathogens.

[164] Hornok S, Kartali K, Takács N, Hofmann-Lehmann R (2016). Uneven seasonal distribution of *Babesia canis* and its two 18S rDNA genotypes in questing *Dermacentor reticulatus* ticks in urban habitats. Ticks Tick Borne Dis.

[165] Wójcik-Fatla A, Bartosik K, Buczek A, Dutkiewicz J (2012). *Babesia microti* in adult *Dermacentor reticulatus* ticks from Eastern Poland. Vector-Borne Zoonotic Dis.

[166] Corduneanu A, Ursache TD, Taulescu M, Sevastre B, Modrý D, Mihalca AD (2020). Detection of DNA of *Babesia canis* in tissues of laboratory rodents following oral inoculation with infected ticks. Parasit Vectors.

[167] Rar VA, Fomenko NV, Dobrotvorsky AK, Livanova LL, Rudakova SA, Fedorov EG (2005). Tickborne pathogen detection, western Siberia, Russia. Emerg Infect Dis.

[168] Majláthová V, Majláth I, Víchová B, Gul'ová I, Derdáková M, Sesztáková E, Pet'ko B (2011). Polymerase chain reaction confirmation of *Babesia canis canis* and *Anaplasma phagocytophilum* in dogs suspected of babesiosis in Slovakia. Vector Borne Zoonotic Dis.

[169] Duh D, Slovák M, Saksida A, Strasek K, Petrovec M, Avšič-Županc T (2006). Molecular detection of *Babesia canis* in *Dermacentor reticulatus* ticks collected in Slovakia. Biologia.

[170] Karbowiak G, Vichová B, Slivinska K, Werszko J, Didyk J, Peťko B (2014). The infection of questing *Dermacentor reticulatus* ticks with *Babesia canis* and *Anaplasma phagocytophilum* in the Chernobyl exclusion zone. Vet Parasitol.

[171] Abdallah OM, Niu Q, Yu P, Guan G, Yang J, Chen Z (2016). Identification of piroplasm infection in questing ticks by RLB: a broad range extension of tick-borne piroplasm in China?. Parasitol Res.

[172] Shock BC, Moncayo A, Cohen S, Mitchell EA, Williamson PC, Lopez G (2014). Diversity of piroplasms detected in blood-fed and questing ticks from several states in the United States. Ticks Tick Borne Dis.

[173] Li LH, Wang JZ, Zhu D, Li XS, Lu Y, Yin SQ (2020). Detection of novel piroplasmid species and *Babesia microti* and *Theileria orientalis* genotypes in hard ticks from Tengchong County, southwest China. Parasitol Res.

[174] Zhuang L, Du J, Cui XM, Li H, Tang F, Zhang PH (2018). Identification of tick-borne pathogen diversity by metagenomic analysis in *Haemaphysalis longicornis* from Xinyang, China. Infect Dis Poverty.

[175] Niu Q, Liu Z, Yang J, Gao S, Pan Y, Guan G (2017). Genetic characterization and molecular survey of *Babesia* sp. Xinjiang infection in small ruminants and ixodid ticks in China. Infect Genet Evol.

[176] Brinkmann A, Hekimoğlu O, Dinçer E, Hagedorn P, Nitsche A, Ergünay K (2019). A cross-sectional screening by next-generation sequencing reveals *Rickettsia*, *Coxiella*, *Francisella*, *Borrelia*, *Babesia*, *Theileria* and *Hemolivia* species in ticks from Anatolia. Parasit Vectors.

[177] Harrus S, Perlman-Avrahami A, Mumcuoglu KY, Morick D, Eyal O, Baneth G (2011). Molecular detection of *Ehrlichia canis*, *Anaplasma bovis*, *Anaplasma platys*, Candidatus* Midichloria mitochondrii* and* Babesia canis vogel*i in ticks from Israel. Clin Microbiol Infect.

[178] Masatani T, Hayashi K, Andoh M, Tateno M, Endo Y, Asada M (2017). Detection and molecular characterization of *Babesia*, *Theileria*, and *Hepatozoon* species in hard ticks collected from Kagoshima, the southern region in Japan. Ticks Tick Borne Dis.

[179] Wattanamethanont J, Kaewthamasorn M, Tiawsirisup S (2018). Natural infection of questing ixodid ticks with protozoa and bacteria in Chonburi Province, Thailand. Ticks Tick Borne Dis.

