# Supplementary material for: The specificity of Babesia-tick vector interactions: recent advances and pitfalls in molecular and field studies
Source: Parasit Vectors. 2021 Sep 28;14:507. doi: 10.1186/s13071-021-05019-3 (PMC8480096; doi:10.1186/s13071-021-05019-3)
Supplement: Supplementary file 1 — Additional file 1: Text S1. Range of this review. [file 13071_2021_5019_MOESM1_ESM.docx]

**Additional file 1: Text S1.** The range of the current review

This review was performed to test the hypothesis that recent eco-epidemiological studies, based on molecular identification of *Babesia* in questing ticks, helped to recognize and confirmed the existence of a specific interaction between certain piroplasm species and certain tick species. Repeated observation of certain *Babesia* species in certain tick species/genus in numerous independent studies, different regions and years, has been considered as epidemiological evidence for established *Babesia*- tick species interaction.

Data were retrieved: i) from original articles published in the period 2000-2021, reporting detection and identification of *Babesia* species/strain in host-seeking ticks (129 original papers examined) and ii) from the GenBank data base on selected *Babesia* species originated from questing ticks of determined species. As the majority of the molecular studies on piroplasm are conducted on 18S rDNA (*ssu*), this genetic marker was analysed in the current review. In the case of suspicious records from GenBank, publication status for certain research has been checked and unpublished results removed from the analysis.

Initially, 168 papers were identified. After initial screening, 39 papers were excluded from analysis due to: i) reporting *Babesia* detection in ticks collected from animals; ii) repetition of results- mainly papers involved in [21]; iii) papers reporting null prevalence of *Babesia* spp.; iv) or without identification of babesiae/tick species. Finally, 129 papers were included in the analyses (Tables 1-3).
