# Supplementary material for: The specificity of Babesia-tick vector interactions: recent advances and pitfalls in molecular and field studies
Source: Parasit Vectors. 2021 Sep 28;14:507. doi: 10.1186/s13071-021-05019-3 (PMC8480096; doi:10.1186/s13071-021-05019-3)
Supplement: Supplementary file 2 — Additional file 2: Table S1. Species of Babesia reported in Ixodes spp. [file 13071_2021_5019_MOESM2_ESM.docx]

Additonal file 2: Table S1. Species of *Babesia* reported in *Ixodes* spp.

| **Country** | **Reference** | ***Ixodes* species (*n*)** | ***Babesia* spp. prevalence** | **Species of *Babesia,* number of isolates and prevalence (%)** | **Species identification method** |
| --- | --- | --- | --- | --- | --- |
| Austria | Blaschitz et al. [89] | *Ixodes ricinus* (853) | 51% (30% in adults; 49% in nymphs and 55% in larvae) | *Babesia divergens- B. divergens-*like (428)*:  *B. divergens*, 6 | PCR-sequencing (selected products sequences, no prevalence calculated) |
|  |  |  |  | *Babesia* sp. strain DD, 3 |  |
|  |  |  |  | *B. venatorum,* 5 |  |
| Belarus | Reye et al. [27] | *Ixodes ricinus* (453) | 1.1% | *B. microti*, 3 (0.7%) | PCR-sequencing |
|  |  |  |  | *B. venatorum*, 2 (0.4%) |  |
| Belgium | Lempereur et al. [90] | *Ixodes ricinus* (805) | 14.6% in feeding  7.9% in questing | *B. venatorum*, 55 | PCR-sequencing |
| Czech Republic | Václavík et al. [91] | *Ixodes ricinus* (13340) | 1.1% (MIR) | *B. venatorum*, 12 | PCR-sequencing |
| Czech Republic | Venclíková et al. [92] | *Ixodes ricinus* (1473) | 0.5% (MIR) | *B. venatorum*, 2 | PCR-sequencing |
|  |  |  |  | *B. capreoli,* 1 |  |
| Czech Republic | Rudolf et al. [93] | *Ixodes ricinus* (350) | 1.4% (MIR) | *B. microti*, 5 pools | PCR-sequencing |
| Czech Republic, Slovakia | Rybarova et al. [54] | *Ixodes ricinus* (1408) | 1.6% | *B. venatorum*, 2 (0.1%) | PCR-sequencing |
|  |  |  |  | *Babesia* sp. (*canis*-like)  20 (1.5%) |  |
| Denmark | Klitgaard et al. [94] | *Ixodes ricinus* (1013) | 0.5% | *B. venatorum*, 5 (0.5%) | qPCR; probes for *B. microti, B. canis, B. venatorum* and *B. divergens* |
| Estonia | Katargina et al. [47] | *Ixodes persulcatus* (938) | 1.4% (*I. persulcatus+ I. ricinus*) | *B. microti* US type, 2 | RLB, PCR-sequencing |
|  |  |  |  | *B. divergens*-like, 2 |  |
|  |  | *Ixodes ricinus* (1665) |  | *B. microti* US type, 6 |  |
| Finland | Sormunen et al. [95] | *Ixodes ricinus* (415) | 1.4% | *B. venatorum*, 6 (1.4%) | PCR-sequencing |
| Finland | Sormunen et al. [96] | *Ixodes ricinus* (7070) | 1.1-1.3% | *B. venatorum*, 22 | PCR-sequencing |
| France | Akl et al. [97] | *Ixodes ricinus* (696) | 0.4% | *B. venatorum*, 3 (0.4%) | PCR-sequencing |
| France | Bonnet et al. [98] | *Ixodes ricinus* (1487) | nd | *B. microti*, 5 | NGS |
|  |  |  |  | *B. venatorum*, 4 |  |
|  |  |  |  | *B. major*-like, 1 |  |
| France | Bonnet et al. [68] | *Ixodes ricinus* (45) | 6.7% | *B. divergens* , 2 (4.4%) | PCR-RLB for selected *Babesia* species |
| France | Jouglin et al. [99] | *Ixodes ricinus* (2620) | 0.6% | *B. venatorum*, 13 (0.5%) | PCR-sequencing |
|  |  |  |  | *B. capreoli*, 2 (0.08%) |  |
| France | Lebert et al. [100] | *Ixodes ricinus* (4518) | 2.9% | *B. venatorum*, 36 | PCR-sequencing |
|  |  |  |  | *B. capreoli*, 2 |  |
| France | Paul et al. [101] | *Ixodes ricinus* (259) | 2% | *B. venatorum* | PCR-sequencing |
|  |  |  |  | *B. divergens* |  |
| France | Reis et al. [102] | *Ixodes ricinus* (227) | 1.3% | *B. venatorum*, 6 (1.3%) | PCR-sequencing |
| France | Lejal et al. [103] | *Ixodes ricinus* (60) | 10% | *B. venatorum*, 6 (10%) | qPCR in different tick organs |
| Germany | Franke et al. [104] | *Ixodes ricinus* (196) | 10.7% | *B. microti*, 10 (5.1%) | PCR- sequencing (no data on sequences) |
|  |  |  |  | *B. divergens*, 11 (5.6%) |  |
| Germany | Franke et al. [105] | *Ixodes ricinus* (293) | 8.9% | *B. microti*-like, 23 (7.8%) | PCR- sequencing (no data on sequences) |
|  |  |  |  | *B. divergens*-like, 3 (1%) |  |
| Germany | Galfsky et al. [106] | *Ixodes ricinus* (547) | 0.4% | *B. microti*, 1 (0.2%) | PCR-sequencing |
|  |  |  |  | *B. venatorum*, 1 (0.2%) |  |
| Germany | Hildebrandt et al. [107] | *Ixodes ricinus* (1000) | 5% | *B. microti*, 18 | PCR-sequencing |
|  |  |  |  | *B. divergens*, 12 |  |
| Germany | Hildebrandt et al. [108] | *Ixodes ricinus* (196) | 10.7% | *B. microti*, n=29 | PCR-sequencing |
|  |  |  |  | *B. divergens*, n=33 |  |
| Germany | Overzier et al. [109] | *Ixodes ricinus* (4381) | 1% | *B. venatorum*, 27 (0.6%) | PCR-sequencing |
|  |  |  |  | *B. microti*, 12 (0.3%) |  |
|  |  |  |  | *B. capreoli*, 6 (0.1%) |  |
| Germany | Overzier et al. [110] | *Ixodes ricinus* (199) | 3% | *B. microti*, 3 (1.5%) | PCR-sequencing |
|  |  |  |  | *B. venatorum*, 2 (1.5%) |  |
|  |  |  |  | *B. capreoli*, 1 (0.5%) |  |
| Germany | Silaghi et a. [43] | *Ixodes ricinus* (782) | 4.1% | *B. microti*, 25 (3.2%) | PCR-sequencing |
|  |  |  |  | *B. venatorum*, 6 (0.8%) |  |
|  |  |  |  | *B. divergens*, 2 (0.3%) |  |
|  |  |  |  | *B. capreoli*, 1 (0.1%) |  |
| Germany | Springer et al. [111] | *Ixodes ricinus* (1430) | 1% | *B. microti*, 7 (0.5%) | PCR-sequencing |
|  |  |  |  | *B. venatorum*, 6 (0.4%) |  |
|  |  |  |  | *B. capreoli*, 1 (0.1%) |  |
| Germany | Schorn et al. [112] | *Ixodes ricinus* (6593) | 0.4% | *B. venatorum*, 25 (0.4%) | PCR-sequencing |
|  |  |  |  | *B. divergens*, 1 (0.02%) |  |
|  |  |  |  | *B. divergens/capreoli*, 1 (0.02%) |  |
|  |  |  |  | *B. gibsoni-*like, 1 (0.02%) |  |
| Germany | Eshoo et al. [113] | *Ixodes ricinus* (226) | 3.5% | *B. microti*, 8 (3.5%) | PCR-sequencing |
| Germany | Hartelt et al. [114] | *Ixodes ricinus* (3113) | 1% | *B. divergens*, 28 (0.9%) | PCR-sequencing |
|  |  |  |  | *B. microti*, 3 (0.1%) |  |
| Germany | Hildebrandt et al. [115] | *Ixodes ricinus* (1000) | 5% | *B. microti*, 28 (2.8%) | PCR-sequencing |
|  |  |  |  | *B. divergens*, 20 (2%) |  |
| Hungary | Egyed et al. [116] | *Ixodes ricinus* (1800) | 0.8% | *B. divergens* (0.5% MIR) | PCR- DNA hybridization |
|  |  |  |  | *B. microti* (0.3% MIR) |  |
| Italy | Aureli et al. [117] | *Ixodes ricinus* (316 samples, including pools) | 4.1% samples | *B. venatorum*, 11 (3.5% samples) | PCR-sequencing |
|  |  |  |  | *B. divergens/capreoli*, 2 (0.6% samples) |  |
| Italy | Capelli et al. [118] | *Ixodes ricinus* (191) | 0.5% | *B. venatorum*, 1 (0.5%) | PCR-sequencing |
| Italy | Cassini et al. [119] | *Ixodes ricinus* (356 in 60 pools) | 0.9% MIR | *B. venatorum* (0.9% MIR) | PCR-sequencing |
| Italy | Floris et al. [120] | *Ixodes ricinus* (1861) | 0.8-1.1% | *B. venatorum*, 12 | PCR-sequencing |
|  |  |  |  | *B. divergens/capreoli*,  2 |  |
| Italy | Zanet et a. [121] | *Ixodes ricinus* (1553 in 161 pools) | 36.7% MIR | *B. venatorum* (23.6% MIR) | PCR-sequencing |
|  |  |  |  | *B. capreoli* (3.1% MIR) |  |
|  |  |  |  | *B. microti* (2.5% MIR) |  |
|  |  |  |  | *B. vulpes* (2.5% MIR) |  |
| Latvia | Capligina et a. [122] | *Ixodes ricinus* (432) | 1.4% | *B. venatorum*, 3 (0.7%) | PCR-sequencing |
|  |  |  |  | *B. microti*, 3 (0.7%) |  |
|  |  | *Ixodes persulcatus* (693) | 1.9% | *B. microti*, 8 (1.2%) |  |
|  |  |  |  | *B. venatorum*, 3 (0.4%) |  |
|  |  |  |  | *B. capreoli*, 2 (0.3%) |  |
| Lithuania and Latvia | Radzijevskaja et al. [123] | *Ixodes ricinus* (370) | 9.5% | *B. microti* Jena, 9 | PCR-sequencing |
|  |  |  |  | *B. venatorum*, 6 |  |
| Luxembourg | Reye et al. [124] | *Ixodes ricinus* (1394) | 2.7% | *B. venatorum*, 22 (1.6%) | PCR-sequencing |
|  |  |  |  | *B. microti*, 13 (0.9%) |  |
|  |  |  |  | *B. divergens*, 1 (0.07%) |  |
| **Netherlands**  **(summary 2000-2019), Belgium** | **Azagi et al. [21]** | ***Ixodes ricinus* (25849)** | **1.9% *Babesia* s.s**. (clade X, [35]) | ***B. venatorum*, 210 (0.8%)** | PCR-RLB, qPCR,  PCR-sequencing |
|  |  |  |  | ***B. capreoli*, 11 (0.04%)** |  |
|  |  |  |  | ***B. divergens*, 4 (0.01%)** |  |
|  |  |  |  | ***Babesia* sp. deer (*B. odocoilei*-like), 1 (<0.01%)** |  |
|  |  | ***Ixodes ricinus* (18626)** | **2.6% *Babesia microti*-like** (clade 1, [35]) | ***B. microti*, 45** | PCR-RLB, qPCR,  PCR-sequencing |
| Norway | Øines et al. [125] | *Ixodes ricinus* (1908) | 0.9% (only *Babesia* s.s.) | *B. venatorum*, 12 (0.6%) | PCR-sequencing for *Babesia* s.s. spp. |
|  |  |  |  | *B. capreoli*, 2 (0.1%) |  |
|  |  |  |  | *B. odocoilei*-like, 1 (0.05%) |  |
| Poland | Cieniuch et al. [55] | *Ixodes ricinus* (1262) | 1.7% | *B. venatorum*, 13 | PCR-sequencing |
|  |  |  |  | *B. canis*, 2 |  |
| Poland | Pieniazek et al. [126] | *Ixodes ricinus* (1328) | 2.1% | *B. divergens*, 26 (2%) | PCR-sequencing |
|  |  |  |  | *B. microti*, 2 (0.2%) |  |
| Poland | Sinski et al. [127] | *Ixodes ricinus* (1513) | 0.6% | *B. microti*, 9 (0.6%) | PCR-sequencing |
| Poland | Stanczak et al. [56] | *Ixodes ricinus* (1875) | 2.5% | *B. venatorum*, 32 (1.7%) | qPCR and nested PCR species/group=specific |
|  |  |  |  | *B. canis*, 13 (0.7%) |  |
|  |  |  |  | *B. divergens*-like, 2 (0.1%) |  |
| Poland | Sytykiewicz et al. [128] | *Ixodes ricinus* (1123) | 3.1% | *B. microti*, 5 | PCR-sequencing |
| Poland | Welc-Faleciak et al. [129] | *Ixodes ricinus* (3165) | 1.6% | *B. microti*, 22 | PCR-sequencing |
|  |  |  |  | *B. venatorum*, 5 |  |
| Poland | Wojcik-Fatla et al. [130] | *Ixodes ricinus* (853) | 4.6% | *B. microti*, 24 (2.8%) | PCR-sequencing |
|  |  |  |  | *B. venatorum*, 10 (1.2%) |  |
|  |  |  |  | *B.* *divergens*, 2 (0.2%) |  |
| Poland | Adamska and Skotarczak [24] | *Ixodes ricinus* (371) | 0.5% | *B. divergens*, 2 (0.5%) | PCR-sequencing |
| Russia | Rar et al. [131] | *Ixodes persulcatus* (789) | 3% | *B. venatorum*, 12 (1.5%) | PCR-sequencing |
|  |  |  |  | *B. microti*, 10 (1.3%) |  |
|  |  |  |  | *B. divergens/B. capreoli*, 2 (0.3%) |  |
| Russia | Rar et al. [40] | *Ixodes persulcatus* (334) | 0.6% | *B. microti* US, 2 (0.6%) | PCR-sequencing |
|  |  | *Ixodes pavlovskyi* (577) | 0.3% | *B. microti* US, 2 (0.3%) |  |
| Russia, Belarus | Movila et al. [132] | *Ixodes ricinus* (297) | 1.7% | *B. venatorum*, 3 (1%) | PCR-sequencing |
|  |  |  |  | *B. microti*, 2 (0.7%) |  |
|  |  | *Ixodes persulcatus* (184) | 0% | - |  |
| Sweden | Karlsson et al. [133] | *Ixodes ricinus* (519) | 4.4% | *B. microti*, 17 (3.2%) | PCR-sequencing |
|  |  |  |  | *B. venatorum*, 5 (1%) |  |
|  |  |  |  | *B. divergens*, 1 (0.2%) |  |
| Serbia | Potkonjak et al. [134]* | *Ixodes ricinus* (71, including 26 from vegetation) | 4.2% | *B. venatorum*, 2 (2.8%) | PCR-sequencing |
|  |  |  |  | *B. microti*, 1 (1.4%) |  |
| Slovakia | Blaňarová et al. [135] | *Ixodes ricinus* (1368) | 0.4% | *B. microti*, 6 (0.4%) | PCR-sequencing |
| Slovakia | Hamšíková et al. [44] | *Ixodes ricinus* (5057) | 1.5% | *B. microti*, 44 (0.9%) | PCR-sequencing |
|  |  |  |  | *B. venatorum*, 26 (0.5%) |  |
|  |  |  |  | *B. capreoli/divergens*  (<0.1%) |  |
|  |  |  |  | *B. canis* (<0.1%) |  |
|  |  |  |  | *B. odocoilei*-like (<0.1%) |  |
| Slovakia | Svehlová et al. [136] | *Ixodes ricinus* (282) | 0.4% | *B. venatorum*, 1 (0.4%) | PCR-sequencing |
| Slovenia | Duh et al. [137] | *Ixodes ricinus* (135) | 9.6% | *B. microti*, 4 | PCR-sequencing |
|  |  |  |  | *B. divergens*-like, 3 |  |
| Spain | Remesar et al. [138] | *Ixodes ricinus* (1056) | 1.8% | *B. venatorum*, 16 (1.5%) | PCR-sequencing |
|  |  |  |  | *B. microti*, 3 (0.3%) |  |
| Spain | Garcia-Sanmartin et al. [53] | *Ixodes ricinus* (193) | 2.5% | *B. bigemina*, 2 (1%) | PCR- RLB |
|  |  |  |  | *B. bovis*, 1 (0.5%) |  |
|  |  |  |  | *B. caballi*, 1 (0.5%) |  |
|  |  |  |  | *B. caballi*-like, 1 (0.5%) |  |
|  |  |  |  | *B. major*, 1 (0.5%) |  |
|  |  |  |  | *B. ovis*, 1 (0.5%) |  |
|  |  |  |  | *B. vulpes* (*T. annae*), 1 (0.5%) |  |
| Switzerland | Burri et al. [139] | *Ixodes ricinus* (465 moulted) | 2.4% | *B. venatorum*, 8 (1.7%) | PCR-RLB |
|  |  |  |  | *B. microti*, 2 (0.4%) |  |
| Switzerland | Casati et al. [140] | *Ixodes ricinus* (1159) | 0.8% | *B. venatorum*, 5 (0.4%) | PCR-sequencing |
|  |  |  |  | *B. microti*, 2 (0.2%) |  |
|  |  |  |  | *B. divergens*-like, 2 (0.2%) |  |
| Switzerland | Gigandet et al. [141] | *Ixodes ricinus* (2568) | 1.7% | *B. venatorum*, 27 (1.1%) | PCR-RLB |
|  |  |  |  | *B. microti*, 13 (0.7%) |  |
|  |  |  |  | *B. divergens*, 4 (0.2%) |  |
| Switzerland | Lommano et al. [142] | *Ixodes ricinus* (1476) | 1.9% | *B. venatorum*, 18 (1.2%) | PCR-RLB |
|  |  |  |  | *B. divergens*, 5 (0.3%) |  |
| Switzerland | Oechslin et al. [143] | *Ixodes ricinus* (1078) | 0.8% | *B. venatorum*, 9 (0.8%) | PCR-sequencing |
| Switzerland | Schaarschmidt et al. [144] | *Ixodes ricinus* (238) | 2.9% | *B. venatorum*, 7 (2.9%) | PCR-sequencing |
| Ukraine | Didyk et al. [145] | *Ixodes ricinus* (696) | 1.9% | *B. microti*, 2 | PCR-sequencing |
| Ukraine | Rogovskyy et al. [146] | *Ixodes ricinus* (280) | 0.4% | *B. microti*, 1 (0.4%) | PCR-sequencing |
| USA | Aliota et al. [147] | *Ixodes scapularis* (922 in 628 pools) | 6% (pools) | *B. microti*, 25 (2.4% MIR) | PCR (species-specific) |
| USA | Edwards et al. [148] | *Ixodes scapularis* (1721) | 3.1% | *B. microti*, 48 (2.8%) | qPCR, PCR-sequencing |
|  |  |  |  | *B. odocoilei*, 5 (0.3%) |  |
| USA | Edwards et al. [149] | *Ixodes scapularis* (423) | 0.7% | *B. microti*, 2 (0.5%) | qPCR, PCR-sequencing |
|  |  |  |  | *B. odocoilei*, 1 (0.25%) |  |
| USA | Hersh et al. [150] | *Ixodes scapularis* (4368) | 12% | *B. microti*, 524 (12%) | PCR (species-specific) |
| USA | Hutchinson et al. [151] | *Ixodes scapularis* (1363) | 3.5% | *B. microti*, 54 (3.5%) | PCR-sequencing |
| USA | Miholland et al. [152] | *Ixodes scapularis* (330) | 6.3% | *B. microti*, 21 (6.3%) | qPCR with TaqMan probes for *B. microti* |
| USA | Prusinski et a. [153] | *Ixodes scapularis* (11184) | 2.5% | *B. microti*, 194 (2.5%) | PCR-sequencing |
| USA | Steiner et al. [154] | *Ixodes scapularis* (394) | 8.6% | *B. odocoilei*, 27 (6.9%) | PCR-sequencing |
|  |  |  |  | *B. microti*, 7 (1.8%) |  |
| USA | Steiner et al. [155] | *Ixodes scapularis* (193) | 11.4% | *B. odocoilei*, 22 (11.4%) | PCR-sequencing |
| Canada | Scott et al. [156] | *Ixodes scapularis* (32) | 12.5% | *B. odocoilei*, 4 (12.5%) | PCR-sequencing |
| Canada | Scott et al. [51] | *Ixodes scapularis* (93) | 4% | *B. odocoilei*, 4 (4%) | PCR-sequencing |
| Canada | Milnes et al. [52] | *Ixodes scapularis* (251) | 1.6% | *B. odocoilei*, 4 (1.6%) | PCR-sequencing |
| Japan | Sivakumar et al. [157] | *Ixodes ovatus* (630) | 0.2% | *B. ovata*, 1 (0.2%) | Species-specific PCR for *Babesia ovata* |
|  |  | *Ixodes persulcatus* (395) | 0% | *-* |  |
| Japan | Zamoto-Niikura et al. [158] | *Ixodes persulcatus* (468) | 3.6% | *B. microti* US, 17 (3.6%) | Strain-specific PCR for *Babesia microti* |
|  |  | *Ixodes ovatus* (315) | 0% | *-* |  |
| Japan | Zamoto-Niikura et al. [48] | *Ixodes persulcatus* (315) | 0.6% | *B. microti* US, 2 (0.6%) | Strain-specific PCR for *Babesia microti* |
|  |  | *Ixodes ovatus* (183) | 21% | *B. microti* Hobetsu, 38 (21%) |  |
| Mongolia | Karnath et al. [159] | *Ixodes persulcatus* (275) | 3.3% | *B. venatorum*, 9 (3.3%) | PCR-sequencing |
| Mongolia | Tuvshintulga et al. [160] | *Ixodes persulcatus* (63) | 30% | *B. microti* Jena-like, 19 (30%) | *B. microti*-specific nested PCR |

* no calculation of prevalence for each species possible; these sequences may encompass also *B. venatorum* and *B. capreoli*

PCR- polymerase chain reaction

qPCR- quantitative PCR

RLB- reverse line blot

NGS- new generation sequencing

MIR- minimal infection rate.
